# FBXO6 regulates colon cancer migration and invasion via ITGB1 ubiquitination and downstream signaling

**DOI:** 10.1038/s41419-026-08554-y

**Published:** 2026-03-19

**Authors:** Niansheng Ren, Long Cheng, Zijian Huang, Xuchen Hu, Fengxu Chi, Yuekun Zhu, Gang Wang

**Affiliations:** 1 Department of Oncology and Laparoscopic Surgery, the First Affiliated Hospital of Harbin Medical University, Harbin, Heilongjiang China; 2 Key Laboratory of Hepatosplenic Surgery, Ministry of Education, The First Affiliated Hospital of Harbin Medical University, Harbin, Heilongjiang China; 3 Department of Colorectal Surgery, the First Affiliated Hospital of Harbin Medical University, Harbin, Heilongjiang China

**Keywords:** Cancer, Immunochemistry, Cancer genomics, Signal transduction

## Abstract

Colorectal cancer (CRC) is the third most common malignant tumor worldwide, with high recurrence and metastasis rates significantly impacting outcomes. This study explores the role of FBXO6, a ubiquitination-related protein, in regulating CRC malignancy, particularly cell migration and invasion. Our analysis reveals that higher FBXO6 expression correlates with better prognosis in CRC patients, although its expression decreases in advanced-stage tumors. Functional studies demonstrate that FBXO6 overexpression suppresses the invasive and migratory abilities of HCT116 and RKO cells and reduces single-cell colony formation. In contrast, FBXO6 knockdown promotes these malignant traits. Immunoprecipitation and mass spectrometry analyses identified ITGB1 as a key substrate of FBXO6, with potential prognostic relevance in CRC. Subsequent in vitro assays confirmed this interaction, revealing that FBXO6 binds ITGB1 at its glycoprotein recognition site, thereby reducing ITGB1 stability and attenuating downstream FAK/PI3K/AKT/ERK signaling. ITGB1 overexpression counteracts the suppressive effects of FBXO6, restoring downstream signaling activity. In vivo xenograft models further validate these findings: FBXO6 overexpression reduces tumor growth, Ki67 levels, and ITGB1-associated signaling. Additional rescue experiments show that FBXO6 counteracts the tumor-promoting effects of ITGB1 overexpression. In conclusion, FBXO6 suppresses CRC cell proliferation, migration, and invasion by targeting ITGB1 for ubiquitination and disrupting key oncogenic signaling pathways, thereby supporting its potential as a prognostic biomarker and candidate therapeutic target in CRC.

## Introduction

Colorectal cancer (CRC) is one of the prevalent malignancies of the digestive system worldwide and represents a significant global public health burden [1]. The disease often presents with subtle signs, making early detection difficult and resulting in many patients being diagnosed at an advanced stage. Globally, approximately 20–25% of CRC patients present with metastases at diagnosis, while another 20–30% of those with locally advanced disease may later progress to metastatic recurrence [2–4]. The 5-year survival rate for metastatic CRC remains below 20% [5]. Despite the evolution of surgical techniques and enhanced clinical diagnostics, the long-term survival rates for patients remain disappointingly low due to the high incidence of recurrence and metastasis [6]. Consequently, developing innovative therapeutic approaches for CRC patients, coupled with a profound understanding of the molecular mechanisms associated with CRC prognosis and relapse, represents a critical and pressing issue that demands immediate attention.

The ubiquitin-proteasome system (UPS) assumes a crucial role in the degradation of proteins within mammalian cells. Composed of ubiquitin (Ub), ubiquitin-activating enzyme (E1), ubiquitin-conjugating enzyme (E2), ubiquitin ligase (E3), and the proteasome, these elements collaborate to enable the recognition, ubiquitination, and subsequent degradation of target proteins [7]. Dysregulation of the UPS disrupts cellular protein stability and contributes to CRC progression [8, 9]. Notably, the malfunction of E3 ubiquitin ligases is frequently associated with the onset and prognosis of CRC, highlighting the significance of maintaining the equilibrium of this system for the preservation of normal cellular functions [10]. Cullin-RING ubiquitin ligases (CRLs), the most extensive E3 ligase family within the UPS, are pivotal in orchestrating protein degradation. The SCF complex, a notable subset of CRLs composed of SKP1, CUL1, and F-box proteins, plays a critical role in substrate recognition, with F-box proteins being the key determinants of substrate specificity. FBXO6, a member of the F-box protein family, is instrumental in managing apoptosis induced by endoplasmic reticulum stress by identifying and degrading misfolded N-glycoproteins and other substrates [11]. Research indicates that FBXO6 may exert a significant influence over the progression of digestive tract cancers. FBXO6 overexpression may promote gastric cancer cell proliferation but reduce invasiveness, possibly by degrading tumor suppressors or stabilizing oncoproteins [12].

Furthermore, emerging evidence suggests that FBXO6 can recognize and ubiquitinate specific protein substrates, thereby regulating cancer-relevant pathways. For instance, in ovarian cancer, FBXO6 mediates the ubiquitination and degradation of RNASET2, promoting tumor progression [13]. Although this mechanism has not been directly demonstrated in CRC, analysis of The Cancer Genome Atlas (TCGA) data indicates that high FBXO6 expression is associated with longer disease-free survival in CRC patients (*P* = 0.0027, hazard ratio = 0.75, 95% CI: 0.62–0.90), suggesting a potential prognostic role for FBXO6 in this cancer type. This substrate-targeting function highlights the possibility that FBXO6 may modulate CRC progression through post-translational mechanisms.

Despite the identified role of FBXO6 in digestive tract cancers, its precise molecular mechanisms in CRC migration and invasion remain unclear. Our study aims to bridge this gap by systematically investigating how FBXO6-mediated ubiquitination governs the aggressive phenotypes of CRC cells. Using immunoprecipitation–mass spectrometry, we identify novel ubiquitination substrates of FBXO6 in CRC and show that this FBXO6–substrate axis drives cell motility and invasion by activating the FAK–PI3K–AKT–ERK signaling cascade. Furthermore, we evaluate the therapeutic potential of targeting FBXO6-dependent pathways to suppress CRC metastasis. This work not only elucidates FBXO6’s regulatory network in CRC but also provides a foundation for developing strategies to inhibit metastatic dissemination.

## Methods

### FBXO6 and prognosis analysis of CRC

The Kaplan–Meier Plotter website (based on the TCGA database, https://kmplot.com) analyzed the prognostic level of FBXO6 in CRC. The GEPIA database (http://gepia.cancer-pku.cn/index.html) analyzed the expression level of the FBXO6 gene in different stages of CRC to indicate the correlation between FBXO6 and the malignancy of cancer.

### Cell culture

Human CRC cell lines HCT116 and RKO were obtained from Zhejiang RuYao Biotechnology Co., Ltd. Upon arrival, cells were thawed in a 37 °C water bath, centrifuged (1000 rpm, 5 min), and resuspended in a complete medium. HCT116 cells were cultured in McCoy’s 5 A medium (Gibco) with 10% FBS (HyClone) and 1% penicillin-streptomycin (Beyotime). RKO cells were maintained in high-glucose DMEM (Gibco) supplemented similarly. Both lines were incubated at 37 °C with 5% CO₂. Medium was replaced every 2–3 days. At 80–90% confluence, cells were passaged using 0.25% trypsin-EDTA (Solarbio) and reseeded at a 1:3–1:5 split ratio. Experiments used cells within 20 passages to maintain consistency.

### Overexpression and silencing of FBXO6 cell construction

The FBXO6 gene sequence (transcript NM_018438.6) was obtained from the NCBI database. The coding sequence (CDS) was synthesized with added EcoRI and NotI restriction sites. The fragment was cloned into the pCDH-CMV-3×Flag-MCS-EF1-COpGFP-T2A-Puro vector using EcoRI and NotI. For FBXO6 knockdown, shRNA primers were designed using the GPP Web Portal (https://portals.broadinstitute.org/gpp/public/). The shRNA sequence was inserted into the pLKO.1 plasmid using BamHI and EcoRI sites. Lentivirus was produced by co-transfecting 293 T cells with the recombinant plasmid, Δ8.91, and pVSV-G (10:10:1 ratio) using lipid-based transfection. After 24 h, the supernatant was collected. HCT116 and RKO cells were infected with the lentivirus in medium containing 2 µg/ml polybrene. After 24 h, the medium was replaced with 2 μg/ml puromycin for selection (7–9 days). Surviving cells were maintained in puromycin-free medium for experiments. FBXO6 overexpression and knockdown efficiency were verified by Western blot at 48 h post-selection.

### qRT-PCR

After extracting total cellular RNA using the Trizol method, 2 μg of mRNA was reverse transcribed into complementary DNA (cDNA) with the 1st Strand cDNA Synthesis Kit gDNA Purge (Novoprotein, China). Quantitative polymerase chain reaction (qPCR) was performed using the SYBR qPCR SuperMix Plus (Novoprotein) and conducted on the 7500 Fast Real-Time PCR System (Applied Biosystems; Thermo Fisher Scientific, Inc.). The relative expression levels of the target gene were determined using the 2^-ΔΔCt^ method. Primers for amplification were designed online through the NCBI website and are as follows: FBXO6-Forward: 5’-TCG GCT GAC TACT TCG TGT TG-3’, FBXO6-Reverse: 5’-TGC TGG AAG AGG ATG TAG CGG A-3’; ITGB1-F: 5’- AGCT GAA GAC TAT CCC ATT GAC C-3’; ITGB1-R: 5’- AAA TGG GCT GGT GCA GTT CT-3’; GAPDH-Forward: 5’-GTC TCC TCT GAC TTC AAC AGC G-3’, GAPDH-Reverse: 5’-ACC ACC CTG TTG CTG TAG CCA-3’.

### Western blot

Cells were lysed with RIPA buffer supplemented with a phosphatase inhibitor cocktail to harvest the proteins. Following SDS-PAGE gel electrophoresis, the proteins were transferred onto a PVDF membrane and blocked with 5% non-fat dry milk for 2 h at room temperature. The primary antibodies [FBXO6 (1:500; ab153853, Abcam), ITGB1 (1;1000; ab52971, Abcam), ITGAV (1:5000; ab179475, Abcam), phospho-FAK (1:1000; ab81298, Abcam; normalized to total FAK), FAK (1:2000; ab40794, Abcam), phospho-PI3K (1:500; PA5-104853, Invitrogen; normalized to total PI3K), PI3K (1:1000; #4292, Cell Signaling Technology), phospho-AKT (1:500; ab38449, Abcam; normalized to total AKT), AKT (1:500; ab8805, Abcam), phospho-ERK (1:1000; ab201015, Abcam; normalized to total ERK), ERK (1:10000; ab184699, Abcam), GAPDH (1:10000; ab181602, Abcam)] were applied and incubated at 4 °C for 16 h. For phosphorylation-specific antibodies (pFAK, pPI3K, pAKT, and pERK), the corresponding total protein (FAK, PI3K, AKT, and ERK) served as loading controls, while GAPDH was used as the loading control for all other proteins. Subsequently, the membranes were incubated with horseradish peroxidase-conjugated goat anti-rabbit or anti-mouse IgG secondary antibodies at a dilution of 1:2000 for 2 h at room temperature. The detection of protein-antibody complexes was achieved using an ECL chemiluminescent substrate, and the ChemiDoc-It Imaging System was utilized for capturing the luminescent signal. GAPDH was employed as an endogenous control for normalization purposes. Quantification of phosphorylated proteins was normalized to their respective total protein levels, while other proteins were normalized to GAPDH. Quantitative analysis of the band intensities was performed using ImageJ software to ensure an accurate statistical assessment of the results.

### Transwell assay

Cells were cultivated to the exponential growth phase, harvested, and washed with PBS to prepare a cell suspension that was adjusted to a concentration of 5 × 10^5^ cells/mL. In the upper chamber of the Transwell apparatus, Matrigel was evenly spread to form a gel layer and was incubated at 37 °C to allow solidification. Subsequently, 200 μL of the adjusted cell suspension was introduced into the upper chamber of the Transwell. In the lower chamber, 600 μL of complete growth medium supplemented with 10% FBS was added. After a 48-hour incubation period, cells that had migrated to the lower surface were fixed with 4% paraformaldehyde and stained with 1% crystal violet. The invasive capacity of the cells was assessed by counting the number of cells in three distinct microscopic fields.

### Wound healing assay

Cells were seeded at a density of 5 × 10^5^ per well in a 6-well plate. Upon reaching 100% confluence, a 200 μL pipette tip was utilized to make a scratch along a straight edge to detach the cells at the scratch. Post-scratch observation and photography were conducted using a phase-contrast microscope, marking this time as 0 h. The cells were then allowed to continue culturing in an incubator, with the migration distance of the marked areas being observed under the phase-contrast microscope at both 0 and 48 h. Ultimately, the migration distances were quantified using the Image-Pro Plus 6.0 software.

### Colony formation assays

Cells were plated at a density of 100 cells per dish with 10 mL of pre-warmed culture medium at 37 °C, ensuring an even distribution by gently swirling the dish. The dishes were then incubated in a cell culture chamber set at 37 °C with 5% CO_2_ and maintained at saturated humidity for 2 to 3 weeks. Once visible colonies were observed with the naked eye, the culture process was halted. The supernatant was removed, and the cells were meticulously rinsed twice using PBS. The cells were fixed with 5 mL of 4% paraformaldehyde for 15 min. Subsequently, an adequate amount of crystal violet staining solution was applied for staining purposes. After photographing the stained colonies, the count of colony formations was conducted using the Image-Pro Plus 6.0 software. The final step involved calculating the total number of colonies formed.

### Immunoprecipitation experiment

Cells were lysed with RIPA buffer to extract proteins. For experimental controls [1]. IgG control: normal rabbit IgG (ab172730, Abcam) was used in place of primary antibody [2]. Blank bead control: protein A/G agarose beads without antibody incubation [3]. Knockout validation: FBXO6-knockout cell lysates were processed in parallel [4]. Positive control: 10% input lysate was reserved prior to immunoprecipitation. Protein A/G agarose beads coupled with a Flag antibody (ab205606, Abcam) were then added and incubated at 4 °C for 6 h to facilitate the binding of immune complexes to the beads. Protein lysate was subsequently added. A volume of 160 μL protein lysate mixed with 40 μL of Loading buffer was heated at 100 °C for 5 min. A subset of the protein samples was reserved for mass spectrometry analysis to identify proteins that co-precipitated with Flag-FBXO6. The enriched proteins were analyzed by Western Blot using specific antibodies against FBXO6, Flag, ITGB1, and GAPDH to determine the expression levels of the target proteins. All controls were processed simultaneously under identical conditions to validate the specificity of the immunoprecipitation results.

### Protein sample preparation for mass spectrometry

Samples stored at −80 °C were thawed and lysed in TEAB buffer (pH ~8.5) using ultrasonication. Protein concentration was determined using a BCA assay kit (P0012, Beyotime) following the manufacturer’s instructions. Equal protein amounts were mixed with 4× loading buffer and 2% SDS (20 μL total volume), then separated by SDS-PAGE at 15 mA (stacking gel) and 35 mA (separating gel) until dye migration was completed. Gels were silver-stained (fixation → sensitization → staining → development for 10 min → termination) for quality assessment. Samples passed if bands were sharp and non-degraded, required re-evaluation if minor smearing/degradation was observed, and were excluded if severely degraded or inconsistent.

### Immunofluorescence

HCT116 and RKO cells were cultured for 24 h to allow for adherence. They were fixed with 4% paraformaldehyde for 20 min to preserve their structure. The cells were permeabilized using 0.1% Triton X-100 to facilitate antibody penetration. After a 20-min blocking step with 1% bovine serum albumin (BSA) to reduce non-specific binding, the cells were incubated with primary antibodies specific to FBXO6 and ITGB1 overnight at 4 °C for 16 h. Negative controls were processed in parallel, omitting primary antibodies and using secondary antibodies only. The primary antibodies were then aspirated, and the cells were incubated with fluorescent secondary antibodies conjugated to FITC, either anti-rabbit or anti-mouse, for 1 h at room temperature. Autofluorescence controls (unstained samples) were included to assess background fluorescence. Following a 10-min DAPI staining to visualize the nuclei (serving as nuclear markers for localization validation), the subcellular localization of the two proteins was examined using a fluorescence microscope. All control samples were processed and imaged under identical conditions as experimental samples to ensure comparability.

### In vitro protein pulldown assay

Building upon the methodology outlined in a previous study [14]. An in vitro co-precipitation experiment was conducted. The FBXO6 gene was cloned into the pET28a-6×His vector. The ITGB1 gene was cloned into the pGEX-4T-1-GST vector. Vector construction was performed by Zhejiang RuYao Biotechnology Co., Ltd. His-FBXO6 and GST-ITGB1 proteins were expressed in E. coli BL21(DE3) and purified. Protein identity was confirmed by LC-MS/MS analysis. For the pulldown assay, 30 μL His-FBXO6 was mixed with 50 μL GST-ITGB1 in 500 μL incubation buffer (50 mM Tris-HCl pH 7.5, 100 mM NaCl, 1 mM EDTA, 0.05% β-mercaptoethanol, 0.2% Triton X-100). The mixture was incubated at 4 °C for 2 h with agitation. 30 μL GST-resin beads were added, followed by incubation at 4 °C for 1 h. The mixture was centrifuged at 4000 rpm. The supernatant was removed. The resin was washed 7–10 times with elution buffer (50 mM Tris-HCl, pH 7.5, 500 mM NaCl, 1 mM EDTA, 0.05% β-mercaptoethanol, 0.2% Triton X-100). Protein interaction was analyzed by Western blot. A control experiment was performed using the GST protein mixed with His-FBXO6 under identical conditions.

### FBXO6 ubiquitination activity site mutation

The ubiquitination activity of FBXO6, which is essential for its binding to glycoproteins, can be suppressed through mutations at the specific sites Y241A and W242A [15]. These mutations were introduced using the QuikChange Lightning Site-Directed Mutagenesis Kit (Catalog no. 210518, Agilent). The mutated sequences were then subcloned into the pCDH-CMV-3×Flag-MCS-EF1-COpGFP-T2A-Puro plasmid. HCT116 or RKO cells overexpressing the mutated FBXO6 were constructed using a lentiviral infection method. Following this, the cells were harvested to assess the expression changes of ITGB1 and the phosphorylation levels of FAK, PI3K, AKT, and ERK proteins.

### In vitro ubiquitination

A mixture was prepared containing 250 ng of Flag-FBXO6 protein and 250 ng of GST-ITGB1 protein, along with 100 ng of E1 (E-305, R&D Systems), 250 ng of E2 (E2-616, R&D Systems), and 2 μg of HA-Ub (U-110, R&D Systems). To this mixture, 2 μL of ubiquitylation buffer (50 mM Tris-HCl, pH 7.5, 5 mM MgCl₂, 2 mM ATP, and 2 mM dithiothreitol [DTT]) was added. The final volume was adjusted to 10 μL with ddH₂O. The reaction mixture was incubated at 30 °C for 3 h. Following the incubation period, 5× SDS-PAGE loading buffer was added to the reaction products, which were then heated at 100 °C for 5 min. The samples were subsequently analyzed by Western blot.

### Analysis of the impact of FBXO6 on the degradation of ITGB1 protein

To investigate the influence of FBXO6 expression levels on the rate of ITGB1 protein degradation, we employed cycloheximide (CHX) and MG132 as analytical tools to assess the protein’s half-life and the pathways involved in its degradation. CHX, serving as an inhibitor of protein synthesis, was utilized to halt the production of new proteins, whereas MG132 functioned as an inhibitor of the proteasome pathway to prevent protein breakdown. The experimental procedure is outlined as follows: HCT116 or RKO cells were exposed to CHX (100 μM) and/or MG132 (25 μM) for durations of 0, 2, 4, and 8 h. Subsequently, the cells were lysed with RIPA buffer, and the expression levels of ITGB1 protein were determined using immunoblotting techniques. Additionally, to elucidate the role of FBXO6 in the stability of ITGB1 protein, we performed knockdown or overexpression of FBXO6 in HCT116 and RKO cells. Afterward, these cells were co-incubated with CHX (100 μM) for the same set of time points, and the expression levels of both FBXO6 and ITGB1 proteins were measured by immunoblotting.

### Rescue experiment

The human ITGB1 coding sequence (CDS, NM_002211.4) was obtained from the NCBI database. An ITGB1 overexpression vector was constructed using pcDNA3.1 as the backbone. HCT116 and RKO cells were seeded in 24-well plates at 50–60% confluency. The cells were transfected with 5 μg of the ITGB1 overexpression plasmid using LipofectamineTM 2000. FBXO6-overexpressing cells or empty vector control cells were specifically targeted. After 48 h, ITGB1 expression was analyzed. qRT-PCR measured mRNA levels. Protein levels were detected by Western blot. Cellular functions were evaluated. Cell proliferation was assessed by CCK-8 assay. Migration was tested using the scratch assay. Invasion was measured by the Transwell assay. The ITGB1/FAK/PI3K/AKT/ERK signaling pathway was examined by Western blot. This provided mechanistic insights into the cellular changes.

### Nude mouse tumor-bearing experiment

Fifteen male nude mice, aged 6 weeks with an average weight of 21 ± 1 g, were randomly assigned into three groups: 1. The Control group; 2. The OverExperssion (OE)-FBXO6 group, where the mice were subcutaneously injected with 5 × 10^6^ HCT116 cells that had been engineered to overexpress FBXO6; 3. The knockdown via shRNA (sh)-FBXO6 group, where the mice received a subcutaneous injection of 5 × 10^6^ HCT116 cells with FBXO6 silenced. Tumor dimensions were recorded at 5-day intervals. At the end of the 25-day experimental period, the mice were humanely sacrificed using CO_2_ asphyxiation. A portion of the tumor tissue was fixed in 4% paraformaldehyde for paraffin embedding. The remaining fresh tissue samples were utilized for immunoblotting analysis to assess the ITGB1 abundance and phosphorylation levels of FAK/PI3K/AKT/ERK.

### Immunohistochemistry

After deparaffinization, tissue sections were immersed in a 0.01 M citrate buffer solution at pH 6.0 and underwent antigen retrieval in a high-pressure steamer at 121 °C for 20 min. Following this, the slides were treated with 3% hydrogen peroxide (H2O2) for 20 min to inactivate endogenous peroxidase activity. They were then incubated with 1% BSA for 20 min at room temperature to block non-specific binding. Subsequently, the slides were incubated with properly diluted primary antibody at 4 °C overnight. Primary antibodies included: FBXO6 (1:100), ITGB1 (1:50), Ki67 (1:50; ab15580, Abcam). The next day, the primary antibody was removed, and the slides were incubated with a biotinylated goat anti-rabbit IgG-HRP secondary antibody for 1 h at room temperature. The color development was performed using a DAB substrate, and the cell nuclei were counterstained with hematoxylin. The slides were examined using a Leica DM500 light microscope, and the immunohistochemical (IHC) results were quantitatively analyzed with Image-Pro Plus 6.0 software.

### Statistical analysis

Data analysis for this study was performed utilizing GraphPad Prism 8.0 software. The normality of the data was first assessed with the Shapiro-Wilk test. Data sets that were normally distributed were expressed as the mean ± standard deviation (Mean ± SD) and analyzed for differences between two groups using the unpaired Student’s *t* test. In cases involving multiple groups, one-way ANOVA with Tukey post hoc analysis was conducted. The significance threshold for all statistical tests was set at *α* = 0.05 (two-tailed).

## Results

### The overexpression of FBXO6 inhibits the colony formation, migration, and invasion abilities of colon cancer cells

**Fig. 1:**
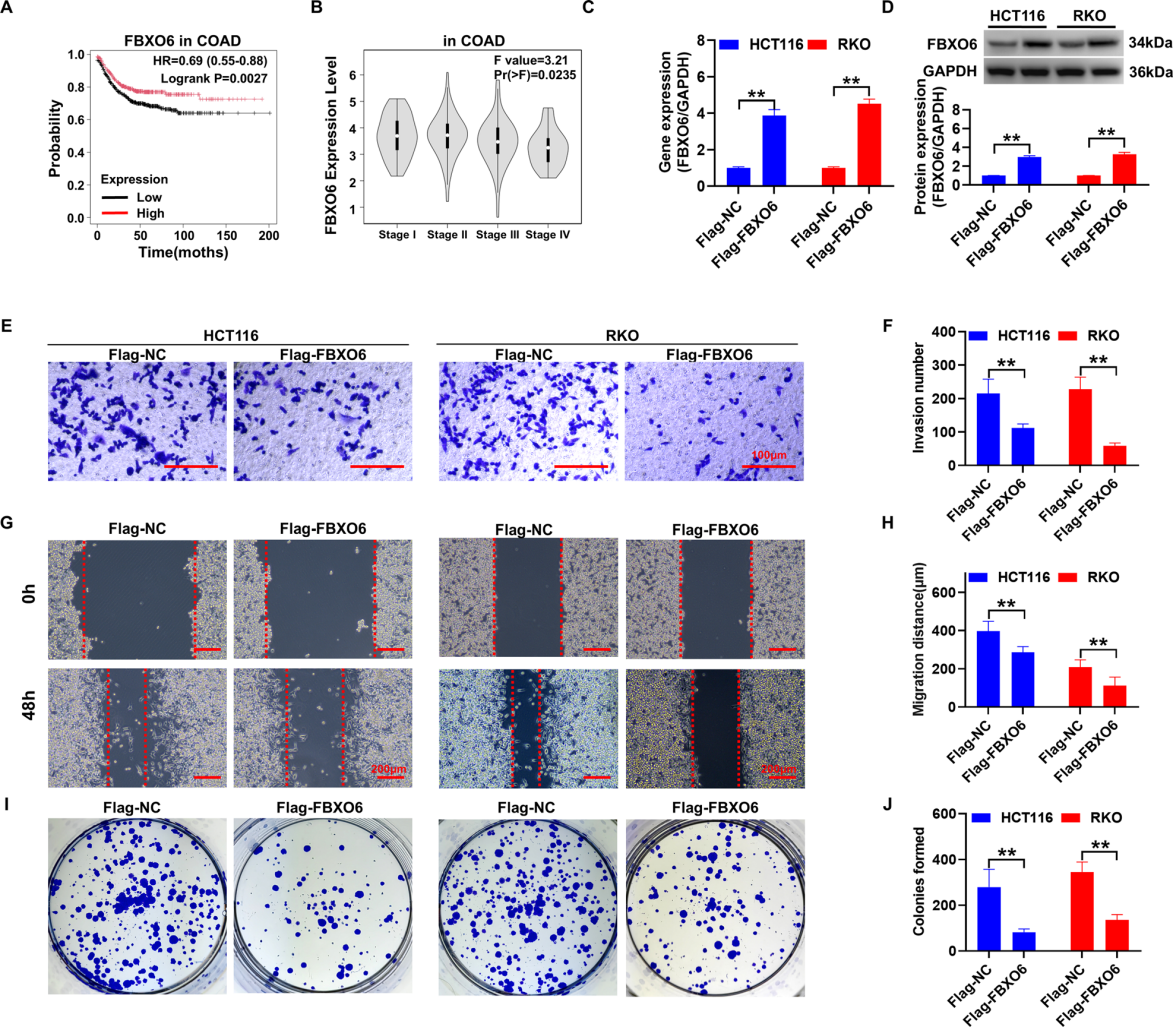
Overexpression of FBXO6 inhibits the proliferation, migration and invasion ability of CRC cells. **A** Kaplan–Meier survival analysis of CRC patients stratified by FBXO6 expression levels (high vs. low) using the KM Plotter database (HR = 0.69, *P* = 0.0027). **B** Stage-dependent FBXO6 expression patterns in CRC from GEPIA analysis, showing a significant reduction in advanced-stage (Stage IV) tumors. Successful FBXO6 overexpression in HCT116 and RKO cells validated by qRT-PCR (**C**) and Western blot (**D**) (*n* = 3 per group). **E**, **F** Transwell invasion assays demonstrating a significant reduction in penetrated cells upon FBXO6 overexpression (*n* = 3 per group), scale bar = 100 μm. **G**, **H** Wound healing assays showing impaired migration capacity in FBXO6-overexpressing cells. Wound closure was monitored at 0 and 48 h (dashed lines indicate initial wound edges; *n* = 3 per group), scale bar = 200 μm. **I**, **J** Colony formation assays revealing suppressed proliferative potential in FBXO6-expressing groups (*n* = 3 per group). Data are presented as mean ± SD. **p *<* 0.01 versus Flag-NC control group. The statistical analysis was performed using an unpaired Student´s *t* test.

The clinical data analysis of the FBXO6 gene in CRC reveals a significant correlation between high FBXO6 expression and improved patient prognosis, with a marked enhancement in survival rates (hazard ratio [HR] = 0.69, *P* = 0.0027, as depicted in Fig. 1A). Furthermore, the FBXO6 gene expression varies significantly across different cancer stages, with a pronounced reduction in expression observed in patients with advanced-stage (Stage IV) disease (F-statistic = 3.21, *P* = 0.0235, Fig. 1B). In cellular studies, both qRT-PCR and Western blot analyses demonstrate a substantial increase in the expression levels of the FBXO6 gene and protein after its overexpression in HCT116 and RKO cell lines, compared to the negative control (NC) group (Fig. 1C, D). Functional assays of the cells show that overexpression of FBXO6 notably suppresses the invasiveness (Fig. 1E, F), migratory ability (Fig. 1G, H), and the capacity for single-cell colony formation on plates (Fig. 1I, J) in HCT116 and RKO cells when contrasted with the control group.

### Silencing FBXO6 promotes the migration, invasion, and single-cell survival capabilities of colon cancer cells

**Fig. 2:**
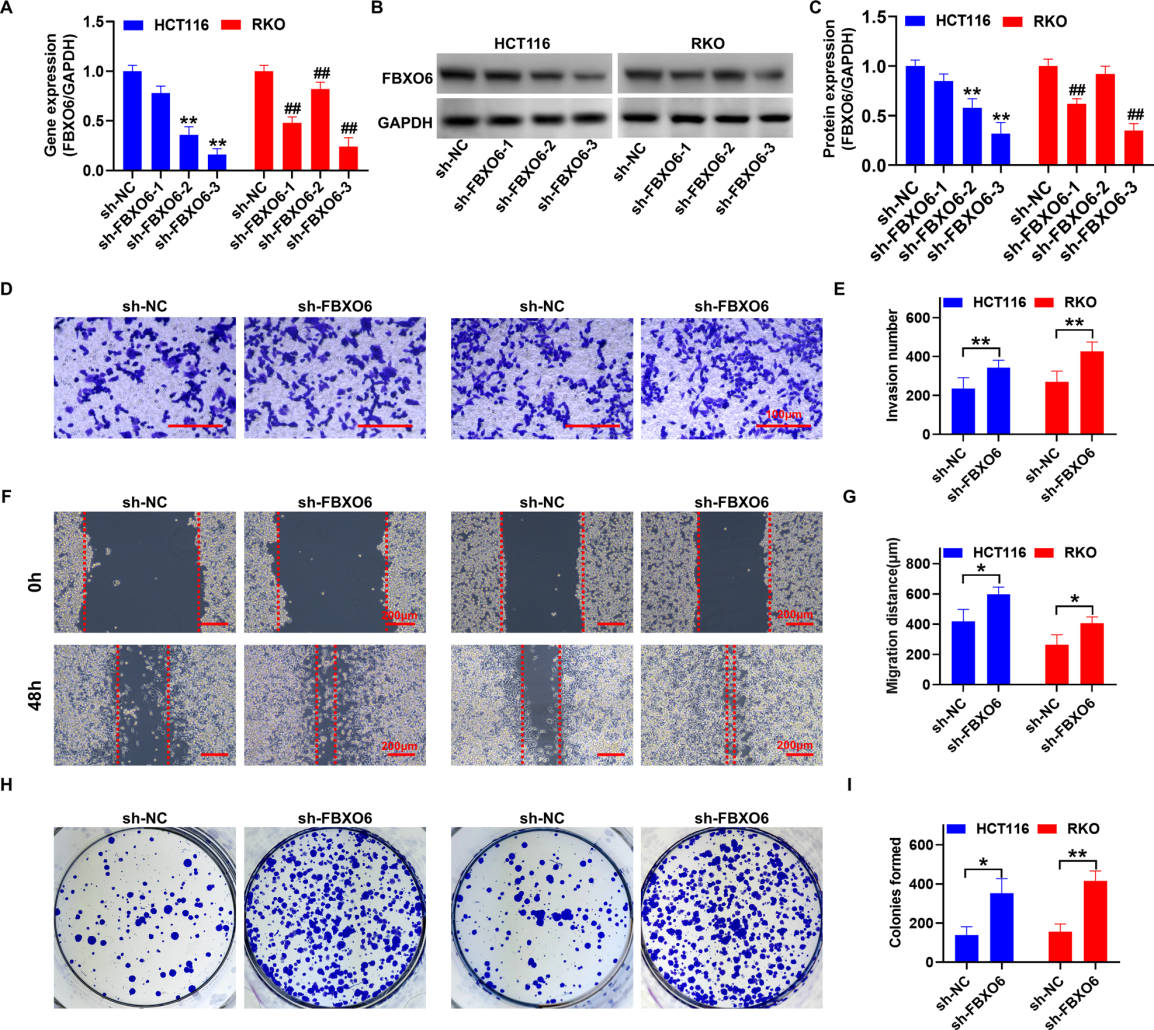
Silencing FBXO6 regulates the migration, invasion, and single-cell survival capabilities of colon cancer cells. **A** Quantitative RT-PCR analysis of FBXO6 mRNA expression levels in HCT116 and RKO cells following shRNA-mediated knockdown (*n* = 3 per group). **B**, **C** Western blot analysis and corresponding densitometric quantification of FBXO6 protein expression in knockdown cells. GAPDH served as a loading control (*n* = 3 per group). **D**, **E** Matrigel invasion assays showing increased invasive capacity upon FBXO6 knockdown. Cells penetrating the Matrigel-coated membranes were stained with crystal violet and quantified from five random fields per condition (*n* = 3 per group), scale bar = 100 μm. **F**, **G** Wound healing assays demonstrating enhanced migratory ability in FBXO6-deficient cells. Wound closure was monitored at 0 and 48 h (dashed lines indicate initial wound edges; *n* = 3 per group), scale bar = 200 μm. **H**, **I** Colony formation assays revealing improved single-cell survival and proliferative capacity after FBXO6 knockdown. Colonies (>50 cells) were counted after 10 days of culture (*n* = 3 per group). Data are presented as mean ± SD. **p < 0.01, *P < 0.05, ##*p* < 0.01 versus sh-NC control group; The statistical analysis was performed using an unpaired Student´s *t* test in (**E**, **G**, **I**). Data in (**A**, **C**) were analyzed using one-way ANOVA with Tukey´s post hoc test.

In this study, we delved deeper into the biological role of FBXO6 in CRC cells by silencing its expression. The qRT-PCR data (Fig. 2A) and immunoblotting analysis (Fig. 2B, C) demonstrate that shRNA3 significantly downregulated the levels of FBXO6 mRNA and protein in HCT116 and RKO cells, with a statistically significant difference from the control group (sh-NC). Subsequent functional assays revealed that the silencing of FBXO6 led to a marked increase in the invasive capacity (Fig. 2D, E) and migratory ability (Fig. 2F, G) of HCT116 and RKO cells. Additionally, the plate colony formation ability of these cells was also significantly enhanced (Fig. 2H, I), indicating that the knockdown of FBXO6 promotes the invasive and proliferative capabilities of CRC cells.

### FBXO6 interactome identifies ITGB1 as a substrate candidate

**Fig. 3:**
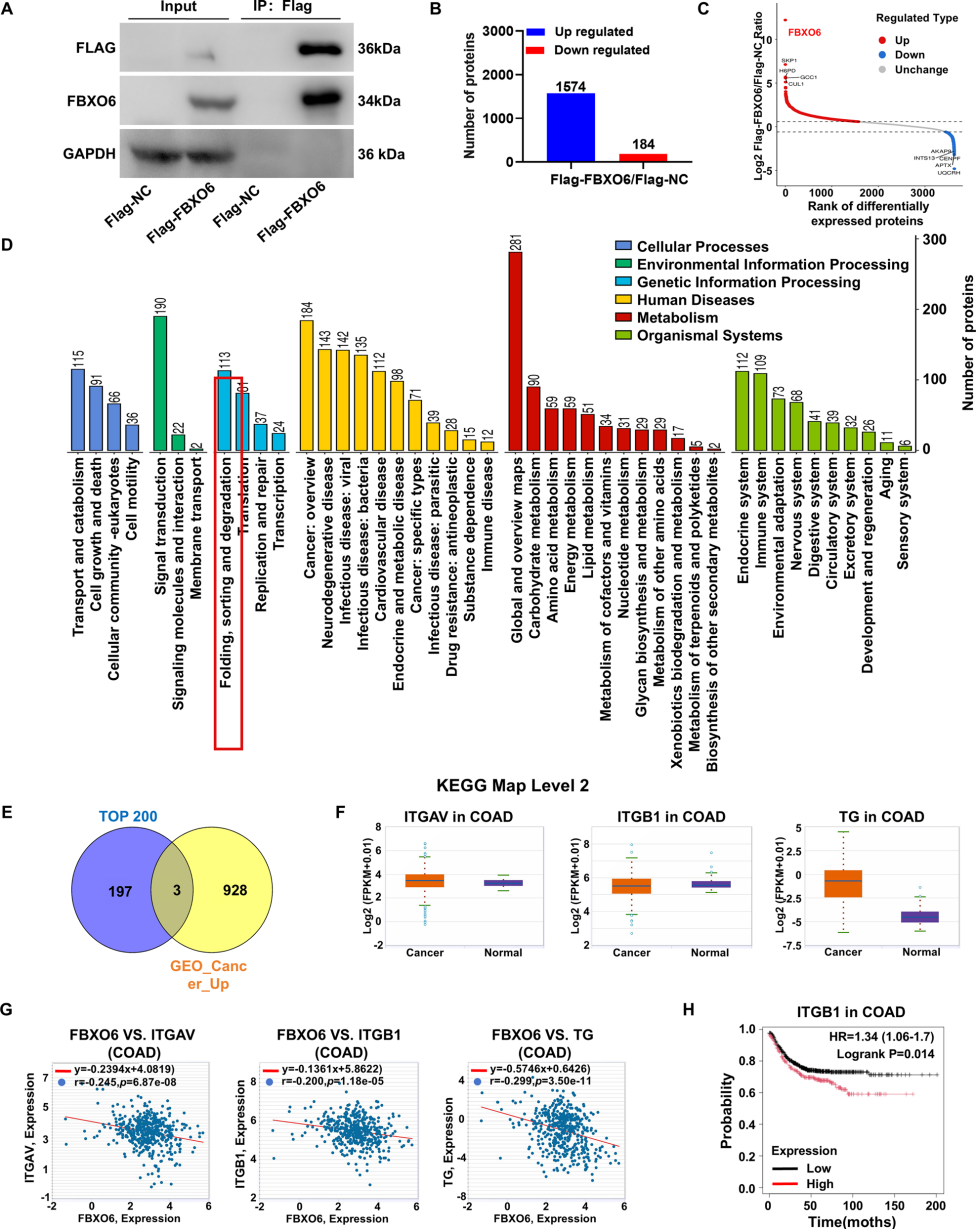
Analysis of the effect of FBXO6 co-immunoprecipitation of ITGB1 protein on the prognosis of CRC. **A** Co-immunoprecipitation (Co-IP) assay demonstrating FBXO6-protein interactions in HCT116 cells transfected with Flag-tagged FBXO6. Immunoprecipitation was performed using anti-Flag magnetic beads. Precipitated proteins were resolved by SDS-PAGE and visualized by silver staining. **B** Quantitative mass spectrometry analysis comparing protein interactions in FBXO6-overexpressing versus control cells. The bar graph shows 1574 enriched (blue) and 184 depleted (red) proteins. **C** Volcano plot of protein interaction changes following FBXO6 overexpression. FBXO6 (red dot) showed the highest enrichment score. **D** KEGG pathway enrichment analysis of FBXO6-bound proteins. **E** Venn diagram intersection analysis identifying three candidate targets (ITGAV, ITGB1, TG). **F** ENCORI database analysis of ITGAV, ITGB1 and TG expression in colon adenocarcinoma (COAD) versus normal tissues. **G** Correlation analysis between FBXO6 and candidate genes in CRC. **H** Kaplan–Meier survival analysis of CRC patients stratified by ITGB1 expression. High ITGB1 expression correlated with worse overall survival.

FBXO6 facilitates the degradation of proteins by ubiquitinating glycoproteins. To identify proteins that directly interact with downstream FBXO6, this study conducted an immunoprecipitation assay of FBXO6 in HCT116 cells. Employing a Flag-tagged FBXO6 construct, we successfully precipitated FBXO6 along with its associated proteins (Fig. 3A). The specificity of the immunoprecipitation was confirmed by silver-stained SDS-PAGE, which showed differential protein bands between Flag-NC and Flag-FBXO6 groups (Supplementary Fig. S1). Mass spectrometry analysis of the precipitated proteins revealed that the overexpression of FBXO6 significantly increased the binding of 1574 proteins, while it decreased for 184 proteins (Fig. 3B). The distribution in the scatter plot indicated that FBXO6 was the predominant protein in the precipitate (Fig. 3C). KEGG enrichment analysis of the upregulated proteins indicated significant regulation of functions related to protein folding, sorting, and degradation (Fig. 3D). A Venn diagram analysis between the top 200 upregulated precipitated proteins and the genes significantly downregulated in CRC cells from the GEO database (GEO ID: GSE160988) identified three proteins that were both upregulated by FBXO6 overexpression and downregulated in CRC: ITGAV, ITGB1, and Thyroglobulin (TG) (Fig. 3E). ENCORI analysis showed no statistically significant differences in the expression of the three genes between COAD and healthy individuals (Fig. 3F). However, there was a significant negative correlation between the gene expression levels of FBXO6 and ITGAV, ITGB1, and TG (Fig. 3G). Further analysis of the prognostic significance of these genes in CRC revealed that high expression of the ITGB1 gene was associated with a poorer prognosis (Fig. 3H). In paired CRC specimens (Supplementary Fig. S2), ITGB1 protein was markedly upregulated (*p* < 0.05), whereas FBXO6 expression remained unchanged. This micro-environment-dependent divergence from the in-vitro inverse pattern underscores ITGB1 as the clinically dominant effector of the FBXO6–ITGB1 axis and suggests additional regulatory inputs acting on FBXO6 activity rather than its abundance.

### FBXO6 can directly bind to ITGB1 and thereby regulate its protein expression level

**Fig. 4:**
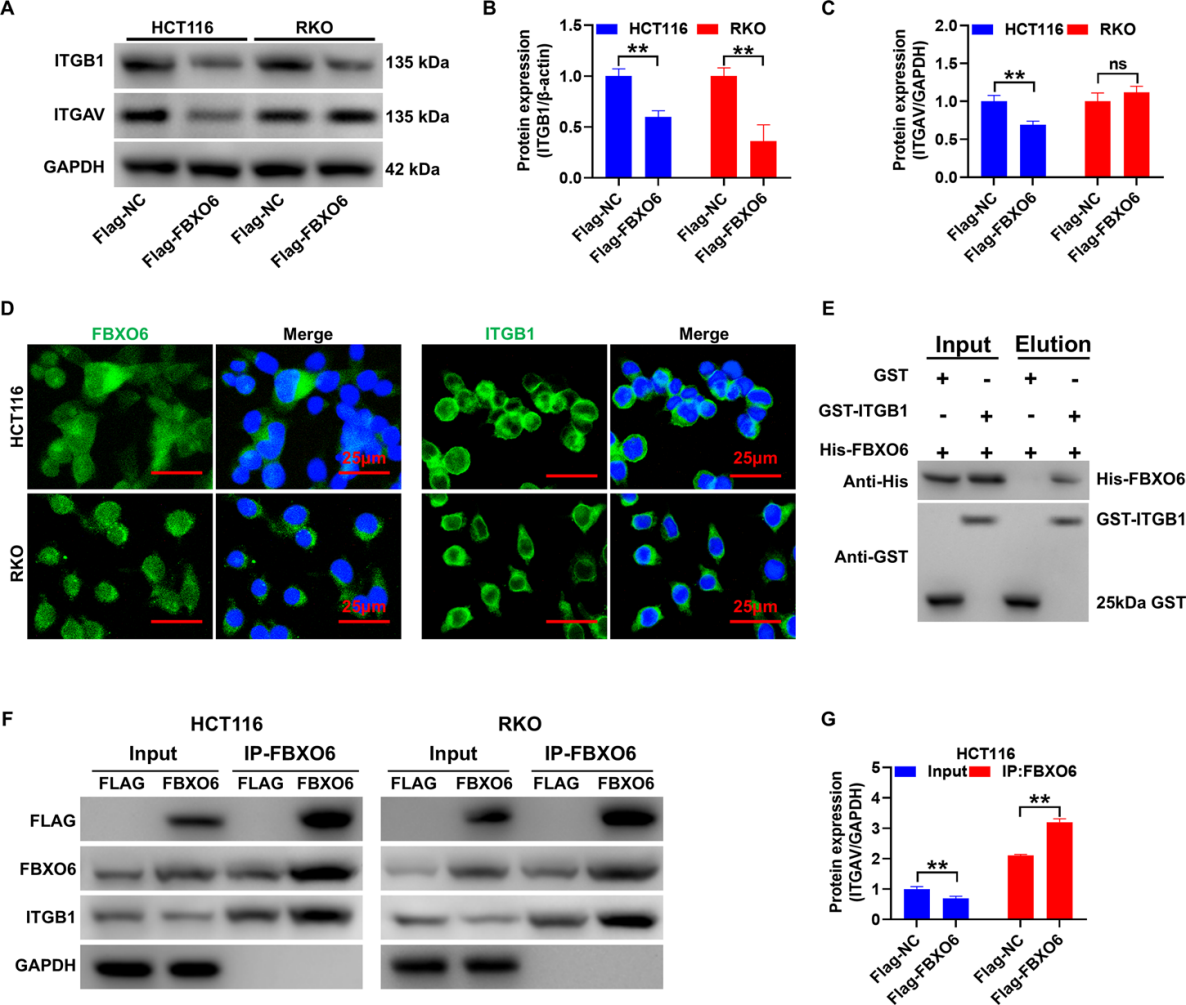
FBXO6 can directly bind to ITGB1 and thereby regulate its protein expression level. **A**–**C** Western blot analysis of ITGB1 and ITGAV expression following FBXO6 overexpression in HCT116 and RKO cells (*n* = 3 per group). ITGAV downregulation was observed in HCT116, but not in RKO. **D** Confocal microscopy showing subcellular localization of FBXO6 (predominantly cytoplasmic) and ITGB1 (cytoplasmic and nuclear membrane). Nuclei were counterstained with DAPI (blue). Scale bar = 25 μm. **E** GST pulldown assay confirming direct interaction between GST–ITGB1 and His–FBXO6. No binding was detected with GST alone. **F**, **G** Co-immunoprecipitation (Co-IP) analysis: IP with anti-Flag beads followed by immunoblotting, FBXO6 overexpression significantly increased ITGB1 binding (*n* = 3 per group). Data are presented as mean ± SD. **p < 0.01, ns(not significant, *p* > 0.05) versus Flag-NC control group. The statistical analysis was performed using an unpaired Student´s *t* test.

To further substantiate the binding interaction between FBXO6 and ITGB1, and to lay the groundwork for the ubiquitination of ITGB1 mediated by FBXO6, this study initially demonstrated at the protein level that the overexpression of FBXO6 notably suppressed the expression of ITGB1 protein in both HCT116 and RKO cell lines. Although the overexpression of FBXO6 also significantly reduced the expression of ITGAV protein, no significant alterations were observed in RKO cells (Fig. 4A–C). Immunofluorescence assays indicated that FBXO6 and ITGB1 are predominantly localized in the cytoplasm, with ITGB1 also showing high expression on the nuclear membrane, suggesting a conducive environment for their interaction. The in vitro pulldown assay revealed the presence of GST-ITGB1 and His-FBXO6 proteins in the Input group. Following the use of glutathione-sepharose beads to adsorb GST, the presence of GST protein was noted after transfection with the GST control and His-FBXO6 plasmids, yet His-FBXO6 protein was not detected with an anti-His antibody. Conversely, after transfection with the GST-ITGB1 and His-FBXO6 plasmids, the GST-ITGB1 protein was detected with an anti-GST antibody, while the GST protein was absent; His-FBXO6 protein expression was identified with an anti-His antibody. These findings confirm the mutual binding capacity between ITGB1 and FBXO6 proteins. Moreover, it is noteworthy that the overall protein levels of ITGB1 were significantly reduced in cells overexpressing FBXO6 (Input group), but there was a marked increase in the expression of adsorbed proteins in the Flag-FBXO6 group following IP-FBXO6 (*p* < 0.01, Fig. 4F, G). This suggests a regulatory role of FBXO6 in the ubiquitination and potentially the degradation of ITGB1. In paired colorectal-cancer specimens, ITGB1 showed a significant increase in protein abundance and IHC H-score relative to adjacent normal mucosa (*p* < 0.05), whereas FBXO6 displayed no significant change at either the mRNA or protein level (*P* > 0.05). These results indicate that ITGB1, rather than FBXO6, is preferentially upregulated in human CRC tissues, underscoring its clinical relevance and hinting at post-transcriptional or activity-based regulation of FBXO6 in the tumor micro-environment (Supplementary Fig. S2).

### FBXO6 mediates the activation of proteins in the FAK/PI3K/AKT/ERK pathway through the activity of glycoprotein binding sites and the expression levels of total proteins

**Fig. 5:**
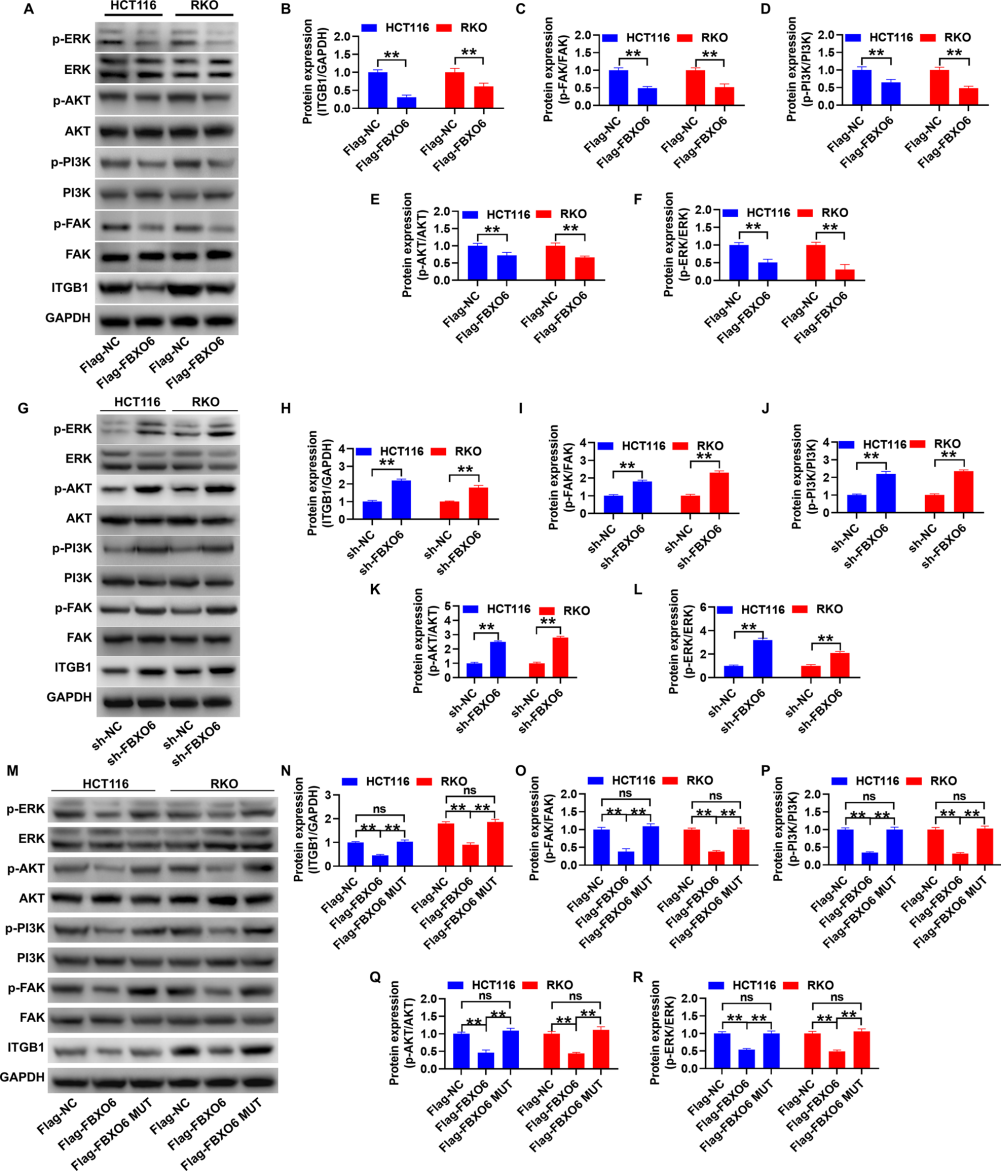
FBXO6 modulates the activation of proteins in the FAK/PI3K/AKT/ERK signaling pathways through the activity of its glycoprotein binding sites and the expression levels of overall proteins. **A**–**F** FBXO6 overexpression effects: Representative immunoblots (top) and densitometric quantification (bottom) showing a significant reduction in ITGB1 protein levels. Phosphorylation decreases: p-FAK, p-AKT, p-ERK. Total protein levels of FAK, PI3K, AKT and ERK remained unchanged (*n* = 3 per group). **G**–**L** FBXO6 knockdown effects: shRNA-mediated FBXO6 depletion caused ITGB1 increases; Phosphorylation increases: p-FAK, p-PI3K, p-AKT, p-ERK, (*n* = 3 per group). **M**–**R** Glycoprotein-binding site mutation (Y241A/W242A): Mutant FBXO6 failed to reduce ITGB1 levels, suppress pathway phosphorylation, and confirms functional requirement of intact glycoprotein-binding domain (*n* = 3 per group). Data are presented as mean ± SD. **p < 0.01, ns (not significant, *p* > 0.05) versus sh-NC or Flag-NC control group; The statistical analysis was performed using an unpaired Student´s *t* test in (**B**–**F**). Data in (**H**–**L**, **N**–**R**) were analyzed using one-way ANOVA with Tukey´s post hoc test.

This section primarily elucidates the impact of FBXO6’s protein regulation on ITGB1 on the critical downstream pathways associated with the prognosis of CRC, specifically the FAK/PI3K/AKT/ERK pathways. Initially, immunoblotting assays demonstrated that the overexpression of FBXO6 markedly suppressed the expression levels of ITGB1 and the phosphorylation of FAK/PI3K/AKT/ERK proteins. When contrasted with the Flag-NC group, these differences were statistically significant (Fig. 5A–F). Conversely, the silencing of FBXO6 led to a significant enhancement in the expression levels of ITGB1 and the phosphorylation of FAK/PI3K/AKT/ERK proteins (Fig. 5G–L). However, upon mutation of the active site amino acids in FBXO6 that interact with glycoproteins, the overexpression of the mutated FBXO6 variant had no significant effect on the expression levels of ITGB1 and the phosphorylation of FAK/PI3K/AKT/ERK proteins. The differences, when compared to the Flag-NC group, were not statistically significant (Fig. 5M–R).

### The impact of FBXO6 on the ubiquitination and degradation levels of ITGB1

**Fig. 6:**
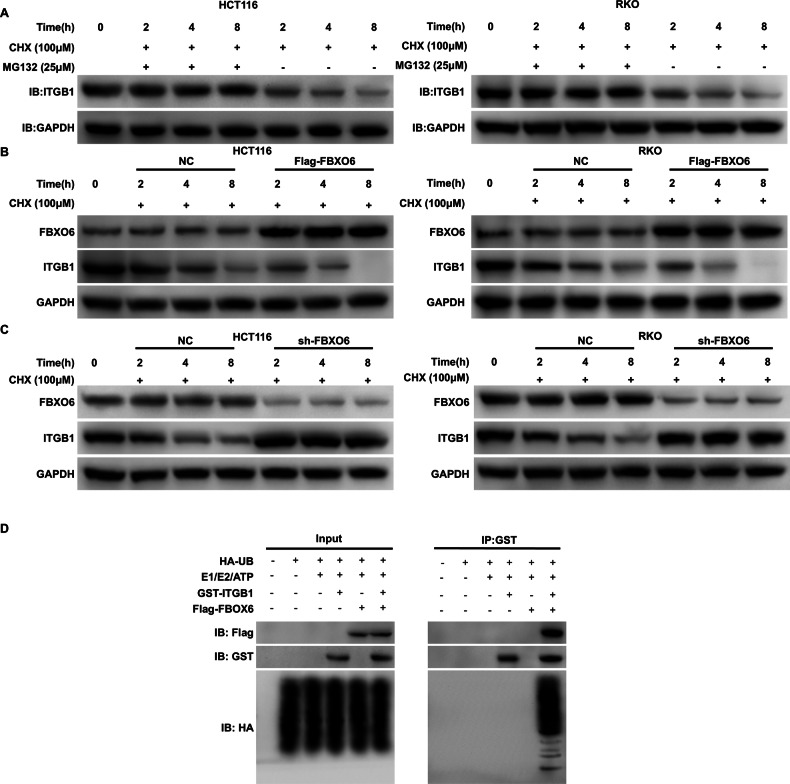
FBXO6 promotes ITGB1 ubiquitination and proteasomal degradation. **A** Proteasome inhibition assay demonstrating ITGB1 stabilization. HCT116 cells were treated with MG132 (25 μM) to block proteasomal degradation. CHX (100 μM) to inhibit new protein synthesis. Immunoblots show ITGB1 protein levels at the indicated time points (0–8 h). Quantification confirms MG132 maintains ITGB1 stability, while CHX treatment leads to progressive decay. **B**, **C** FBXO6 knockdown prolongs ITGB1 stability. **D** In vitro ubiquitination assay. GST pulldown shows FBXO6-dependent polyubiquitination of ITGB1. No ubiquitination signal was detected without FBXO6. Reaction components (UB, E1, E2) are indicated above the lanes.

MG132 is a proteasome inhibitor that impedes the ubiquitin-proteasome pathway of protein degradation; Cycloheximide (CHX) is an antifungal antibiotic that functions as an inhibitor of protein synthesis in eukaryotic organisms, thereby suppressing the synthesis of proteins within the cell. Immunoblotting analysis showed that ITGB1 levels remained stable over 0–8 h when proteasomal degradation was inhibited by MG132, indicating that ITGB1 turnover is largely proteasome dependent. In contrast, treatment with CHX alone led to a time-dependent decline in ITGB1 abundance (Fig. 6A). CHX-chase assays showed that FBXO6 overexpression shortened the half-life of ITGB1 over 0–8 h, whereas FBXO6 knockdown prolonged it (Fig. 6B, C). In vitro ubiquitination assays further demonstrated that FBXO6 is required for ITGB1 ubiquitination: robust polyubiquitinated ITGB1 was detected in the presence of FBXO6, whereas only a background signal was observed in its absence (Fig. 6D; IP, GST group, lane 4).

### FBXO6 regulates the proliferation, migration, and invasion capabilities of CRC through the ITGB1 pathway

**Fig. 7:**
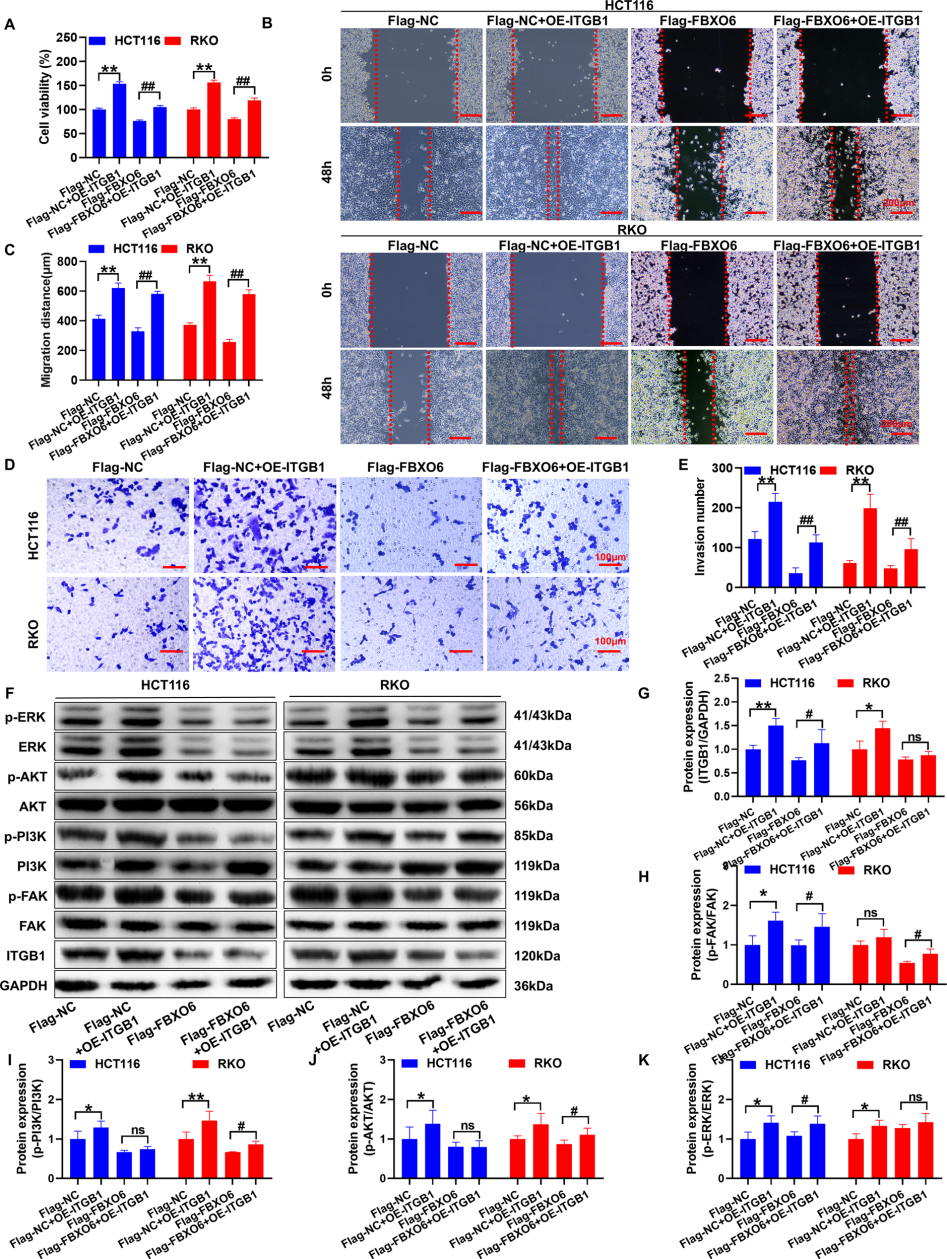
FBXO6 regulates the proliferation, migration, and invasion capabilities of CRC through the ITGB1 pathway. **A** CCK-8 proliferation assay comparing four experimental groups: Flag-NC (empty vector control), Flag-NC + ITGB1 (vector control with ITGB1 overexpression), Flag-FBXO6 (FBXO6 overexpression), and Flag-FBXO6 + ITGB1 (dual overexpression). ITGB1 co-expression significantly rescued the growth-inhibitory effect of FBXO6 (I = 3 per group). **B**, **C** Wound healing assays. Wound closure was monitored at 0 and 48 h (dashed lines indicate initial wound edges; *n* = 3 per group), scale bar = 200 μm. **D**, **E** Matrigel invasion assays (crystal violet staining; *n* = 3 per group), scale bar = 100 μm. **F**–**K** Western blot analysis of: ITGB1/Phospho-FAK/Phospho-PI3K/Phospho-AKT/Phospho-ERK. Densitometric analysis confirmed NC and NC + ITGB1 exhibited comparable protein levels FBXO6 significantly reduced ITGB1 and pathway phosphorylation, ITGB1 co-expression restored pathway activation, (*n* = 3 per group). Data are presented as mean ± SD. *p < 0.05, **p < 0.01, ns (not significant, *p* > 0.05) versus NC control group; #*p* < 0.05, ##*p* < 0.01, ns (not significant, *p* > 0.05) versus FBXO6 control group. The statistical analysis was performed using one-way ANOVA with Tukey´s post hoc test.

The Rescue experiments conducted by overexpressing ITGB1 confirm that FBXO6 modulates tumor biological functions via the ITGB1 pathway. In control experiments, NC (negative control) and NC + ITGB1 groups exhibited baseline proliferation, migration, and invasion levels, which were slightly higher than FBXO6-overexpressing groups but without statistical significance (Fig. 7A–E). The biological function assays demonstrate that the overexpression of ITGB1 significantly enhances the proliferative capacity of CRC cells (Fig. 7A), the distance of cell migration (Fig. 7B, C), and the number of invasive cells (Fig. 7D, E) compared to both NC and FBXO6 groups. Additionally, it upregulates the expression of ITGB1 protein and the phosphorylation levels of FAK/PI3K/AKT/ERK proteins. These changes are statistically significant when compared to the FBXO6 group (Fig. 7F–K), while the NC and NC + ITGB1 groups showed moderately higher but non-significant levels of these proteins compared to FBXO6 alone (Fig. 7F–K).

### FBXO6 regulates the growth of CRC tumors in nude mice by modulating ITGB1/FAK/PI3K/AKT/ERK proteins

**Fig. 8:**
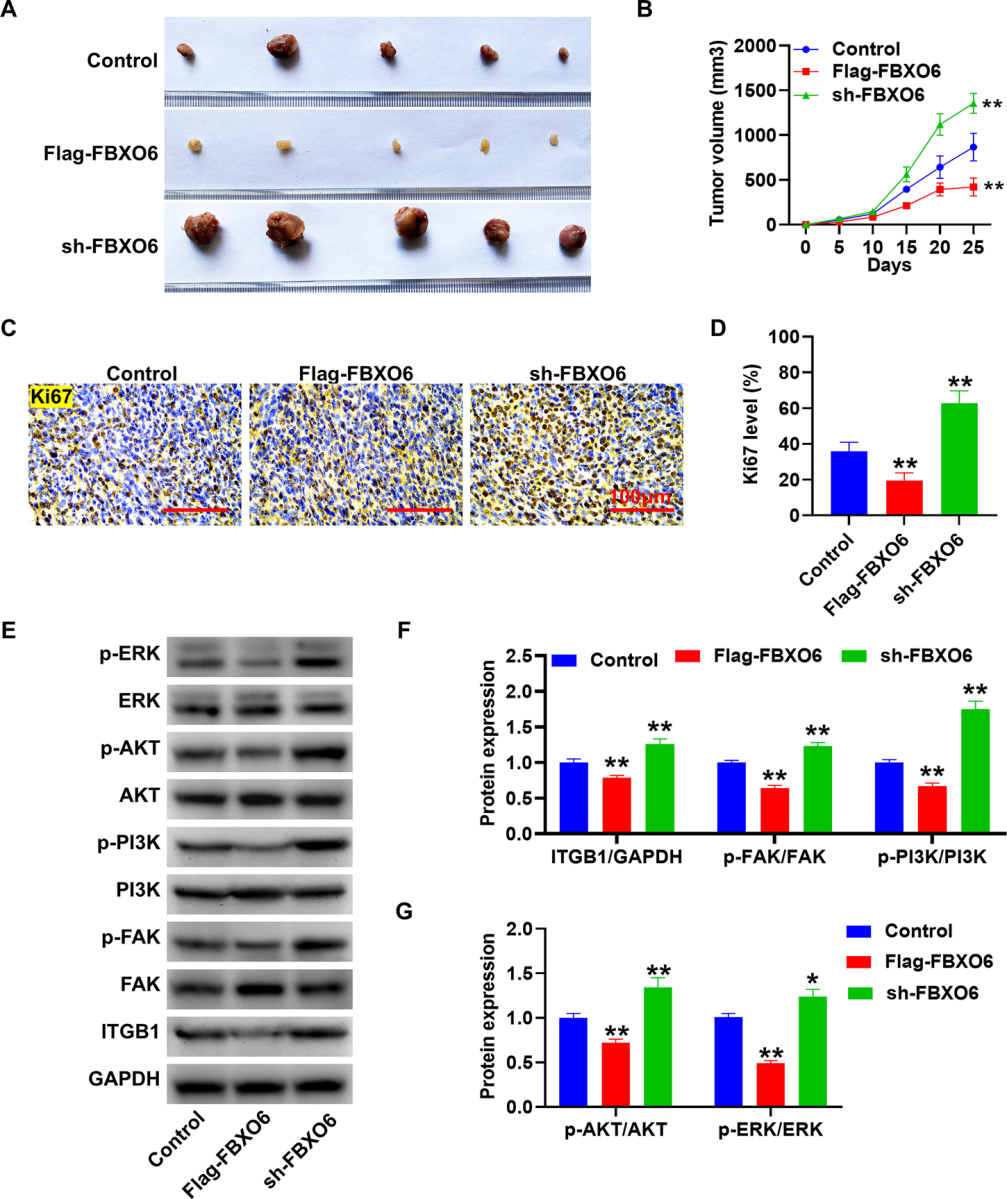
FBXO6 regulates the growth of CRC tumors in nude mice by modulating the ITGB1/FAK/PI3K/AKT/ERK proteins. **A** Representative photograph of subcutaneous xenograft tumors at endpoint (day 28 post-inoculation). FBXO6-overexpressing tumors (Flag-FBXO6 group) showed a significant reduction compared to the control, while FBXO6-knockdown (sh group) tumors exhibited a significant increase. **B** Tumor growth curves measured 5-day intervals (*n* = 5 per group). FBXO6-OE tumors showed significant growth inhibition. **C**, **D** Ki67 immunohistochemistry (IHC) and quantification. The tumors in the Flag-FBXO6 group showed significant reduction in Ki67+ cells, while the sh-FBXO6 group displayed a significant increase (*n* = 3 per group), Scale bar = 100 μm. **E**–**G** Western blot analysis of signaling pathway components: ITGB1/Phospho-FAK/Phospho-PI3K/Phospho-AKT/Phospho-ERK, decrease in Flag-FBXO6 groups, increase in sh-FBXO6 groups. Data are presented as mean ± SD. *p < 0.05, **p < 0.01 versus control group; The statistical analysis was performed using one-way ANOVA with Tukey´s post hoc test.

The tumor volume was markedly decreased following the overexpression of FBXO6, whereas the volume of CRC tumors was significantly enlarged after the silencing of FBXO6. In comparison to the Control group, these differences were statistically significant (Fig. 8A, B). Consistent with the tumor-promoting role of ITGB1, independent modulation of ITGB1 expression (OE-ITGB1 and sh-ITGB1) exerted the expected effects on tumor growth, the co-expression of FBXO6 rescued the ITGB1-mediated effects (Supplementary Fig. S3), further supporting ITGB1 as a critical downstream effector of FBXO6. The Ki67 positivity rate was notably reduced in the FBXO6-overexpressing group and conversely, significantly elevated in the FBXO6-silenced group, with both changes being statistically significant relative to the Control group (Fig. 8C, D). Protein-level validation further confirmed the regulatory role of FBXO6 in the ITGB1 pathway. Western blot analysis showed that FBXO6 over-expression markedly reduced ITGB1 abundance and the phosphorylation of FAK, PI3K, AKT, and ERK relative to the vector control (all *p* < 0.05). In contrast, FBXO6 knockdown (sh-FBXO6) produced the opposite effect, restoring or even elevating ITGB1 and the corresponding phospho-proteins to above control levels (all *p* < 0.05; Fig. 8E–G). These findings substantiate that FBXO6 negatively regulates the ITGB1-FAK/PI3K/AKT/ERK signaling axis in CRC cells.

## Discussion

Our research indicates that FBXO6 regulates the stability of the integrin family member ITGB1 through ubiquitin-proteasomal degradation, uncovering a previously unknown mechanism that controls the invasiveness of CRC cells (Figs. 3–6). While the role of ITGB1 in metastasis is well-documented [16], its posttranslational regulation in CRC had not been explored until now.

The LC-MS/MS screening analysis of FBXO6 immunoprecipitation has pinpointed three proteins potentially implicated in the progression of CRC: ITGAV, ITGB1, and TG. Cancer prognosis databases show that high TG protein levels correlate with better CRC outcomes. This appears contradictory to our finding that FBXO6-mediated TG degradation may suppress CRC progression. Both ITGAV and ITGB1 proteins have been correlated with adverse outcomes in CRC [17]. Both proteins show a negative correlation with FBXO6 expression in CRC. However, cellular assays revealed that FBXO6 overexpression suppressed ITGB1 but not ITGAV in RKO cells. Consequently, ITGB1 is identified as the primary target protein for further investigation into the regulatory effects of FBXO6.

Although FBXO6’s function in protein ubiquitination is known, its impact on FAK/PI3K/AKT/ERK signaling remains undefined. Our integrated findings demonstrate that [1]. FBXO6 physically interacts with ITGB1 (Co-IP/MS in Fig. 3) and promotes its ubiquitin-dependent degradation (Figs. 5, 6) [2]. ITGB1 is a known upstream activator of FAK/PI3K/AKT/ERK cascade, as evidenced by prior studies showing ITGB1 knockdown suppresses phospho-AKT/ERK in CRC [18–20]. and [3]. FBXO6 overexpression significantly reduces ITGB1 protein levels (Figs. 5G, 7F, 8E) and concomitant phosphorylation of FAK/PI3K/AKT/ERK components, which is rescued by ITGB1 co-expression (Fig. 7F). Our results identify FBXO6 as a new negative regulator of FAK/PI3K/AKT/ERK signaling via ITGB1 degradation. Additional FBXO6 substrates from our proteomics analysis (Fig. 3) may also modulate this pathway, requiring further study.

Mook et al. first demonstrated ITGB1’s role in colon cancer liver metastasis using rat models [21]. Clinical studies further show that high ITGB1 expression predicts poor prognosis in CRC patients [22]. Targeted inhibition of ITGB1 can effectively reduce the invasive and migratory capabilities of CRC cells [18]. At the same time, inhibiting the expression of ITGB1 also enhances the tumor-suppressive effect of cetuximab on CRC [23]. ITGB1 activates FAK by promoting phosphorylation at Tyr397. As a key regulator, FAK controls CRC cell survival, migration and metastasis [24–26]. After ITGB1 activates Tyr397 phosphorylation, FAK can activate the P85 subunit of PI3K in CRC cells and the downstream activation of Akt [27, 28]. which is of great significance for activating the malignant characteristics of CRC invasion and metastasis. Therefore, the regulatory mechanism based on the ITGB1 protein helps control the metastasis of CRC and achieve a good prognosis [29].

Based on existing evidence and our data, we propose that FBXO6 acts as a tumor suppressor in CRC. Mechanistically, FBXO6 may downregulate ITGB1 expression, thus inhibiting FAK/PI3K/AKT/ERK signaling and reducing CRC cell motility. However, the precise mechanism by which FBXO6 directly modulates the expression of the ITGB1 protein warrants further investigation. FBXO6 specifically recognizes glycoproteins for ubiquitin-mediated degradation [30]. As ITGB1 is a transmembrane glycoprotein, we speculated that FBXO6 might target ITGB1. In the context of ubiquitination-related experiments, our study has established that ITGB1 is degraded through the ubiquitin-proteasome pathway, and the overexpression of FBXO6 has successfully accelerated the degradation rate of ITGB1. FBXO6, when present, can mediate the ubiquitination of ITGB1. Furthermore, by inducing mutations at the amino acid residues (Y241A and W242A) targeted by FBXO6 for binding to glycoproteins [15]. the binding activity is compromised. Western blot analysis revealed that FBXO6-MUT overexpression failed to alter ITGB1 phosphorylation or downstream signaling. This confirms that FBXO6 requires intact glycoprotein-binding activity to regulate ITGB1. This suggests that FBXO6 targets ITGB1 through its glycoprotein binding capacity, leading to the ubiquitination of ITGB1 and culminating in its degradation.

The current study demonstrates FBXO6-mediated ubiquitination of ITGB1 through co-IP and proteasome inhibition assays (Fig. 6). However, we recognize that future studies should employ ubiquitin linkage-specific mutants (e.g., K48R/K63R) to better characterize the ubiquitination pattern. Three lines of evidence support our conclusions about proteasomal degradation: First, we observed ITGB1 stabilization under MG132 treatment. Second, CHX treatment showed a shortened ITGB1 half-life. Third, prior reports document similar ubiquitination mechanisms [31–33]. Future studies utilizing ubiquitin mutants will be essential to delineate whether FBXO6 exclusively mediates K48-linked polyubiquitination or also participates in non-degradative ubiquitin signaling.

Our large-scale survival analysis identifies FBXO6 as a potential prognostic biomarker in CRC (HR = 0.69, *P* = 0.0027), though its independent predictive value requires validation through multivariate analyses. We acknowledge limitations in the available clinical data. The public datasets (KM Plotter, TCGA) lacked detailed treatment histories and mutational profiles that could affect outcomes. Importantly, our mechanistic findings reveal FBXO6 regulates CRC progression through ITGB1/FAK/PI3K/AKT/ERK axis suppression, suggesting its biological relevance in tumor progression. Future prospective studies with matched clinical variables are warranted to validate FBXO6’s clinical utility.

A limitation of this study is that the initial variability observed between control groups (sh-NC vs. Flag-NC) in experiments such as colony formation assays (Figs. 1I, J and 2H, I) was resolved upon rigorously standardized repetition. We systematically validated these observations and confirmed no significant experimental parameter differences between these control groups (Supplementary Fig. S1B–J). This suggests that the earlier discrepancies likely originated from technical variables, including minor deviations in cell seeding density, asynchronous culture conditions, or inter-batch reagent variations. While these fluctuations did not affect the conclusions, they highlight the critical importance of experimental standardization, particularly for long-term studies involving multiple group comparisons. In subsequent research, we will implement automated cell counting and real-time culture monitoring to minimize operator-dependent variability. These methodological insights provide valuable guidance for similar experimental designs [1]. key phenotypic analyses require validation through independent replicates, and [2]. Control group baseline consistency should serve as a primary reliability criterion for data interpretation.

In conclusion, our research reveals how FBXO6 regulates CRC progression. Specifically, FBXO6 promotes ITGB1 ubiquitination and degradation, thereby inhibiting FAK pathway activation and subsequent PI3K/AKT/ERK signaling. This mechanism explains FBXO6’s role in suppressing cell invasion and migration. This insight provides an important understanding of FBXO6’s potential involvement in CRC progression and prognosis and suggests additional therapeutic avenues for patients with low FBXO6 expression. Since ITGB1 confers drug resistance in tumors, targeting FBXO6 could potentially enhance chemotherapy efficacy. Although we have not directly tested FBXO6’s role in chemoresistance, this represents an important future direction. Linking FBXO6 to drug resistance mechanisms may yield clinically relevant therapeutic strategies.

## Supplementary information

Figure S1

Figure S2

Figure S3

Supplementary Figure legends

Supplemental Material--WB

## Data Availability

The datasets used and/or analyzed during the current study are available from the corresponding author on reasonable request.
