# Supplementary figures and images for: FBXO6 regulates colon cancer migration and invasion via ITGB1 ubiquitination and downstream signaling
Source: Cell Death Dis. 2026 Mar 19;17(1):324. doi: 10.1038/s41419-026-08554-y (PMC13039278; doi:10.1038/s41419-026-08554-y)

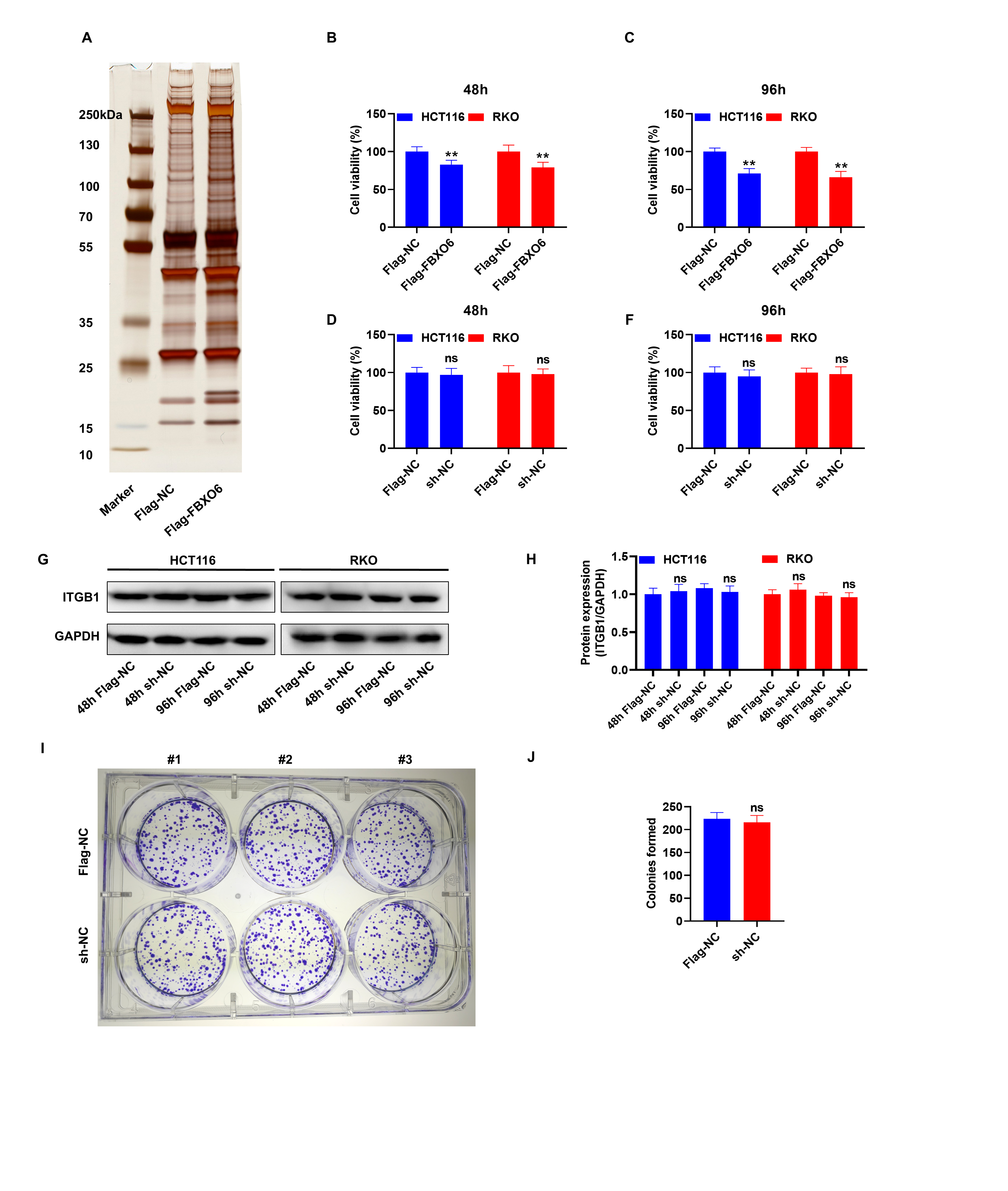

Supplement: Supplementary file 1 — Figure S1 [file 41419_2026_8554_MOESM1_ESM.tif]

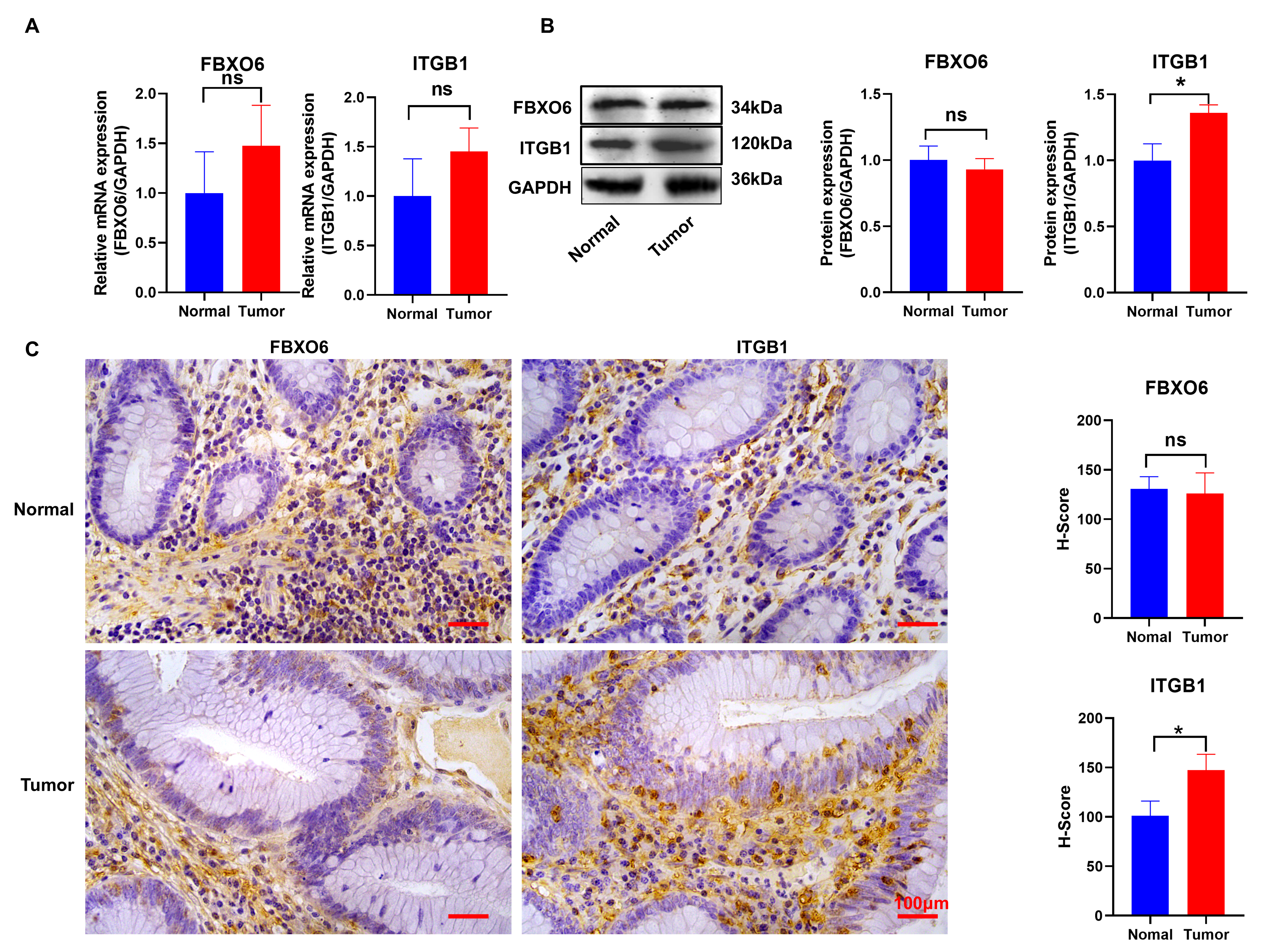

Supplement: Supplementary file 2 — Figure S2 [file 41419_2026_8554_MOESM2_ESM.tif]

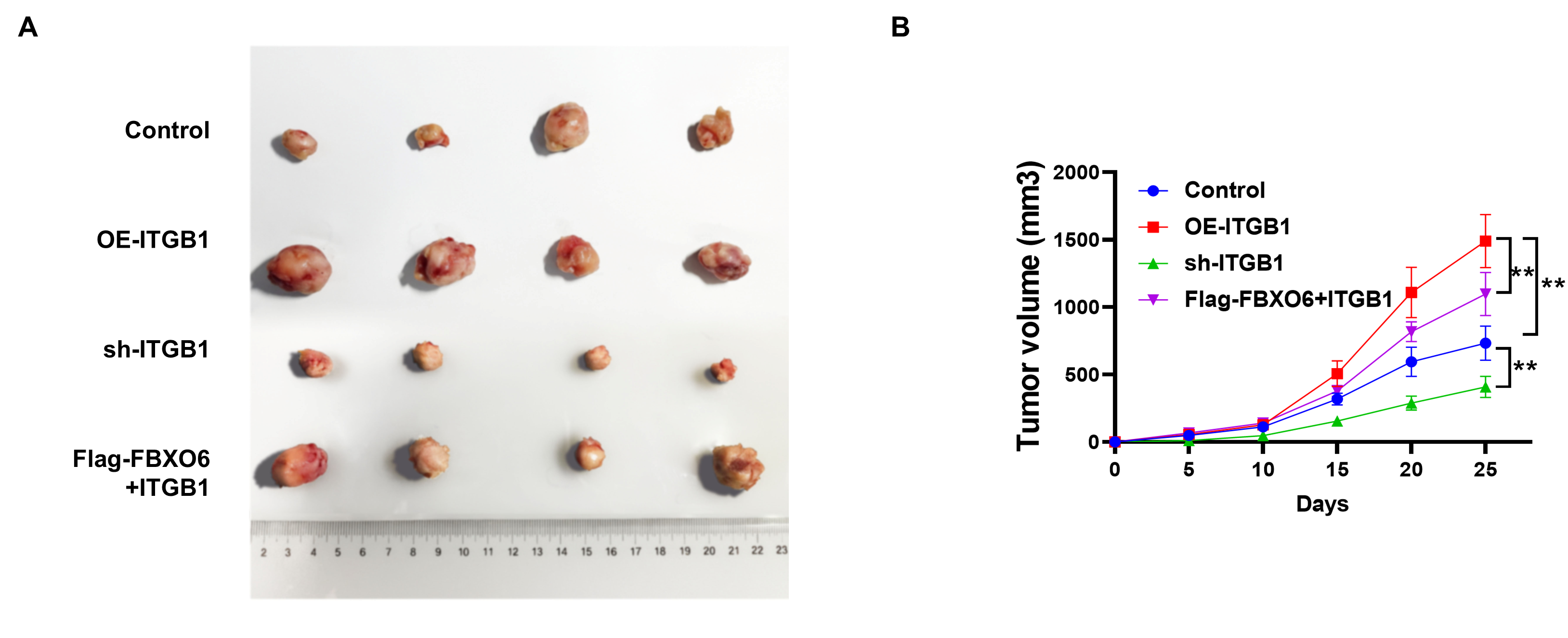

Supplement: Supplementary file 3 — Figure S3 [file 41419_2026_8554_MOESM3_ESM.tif]
