# Supplementary material for: FBXO6 regulates colon cancer migration and invasion via ITGB1 ubiquitination and downstream signaling
Source: Cell Death Dis. 2026 Mar 19;17(1):324. doi: 10.1038/s41419-026-08554-y (PMC13039278; doi:10.1038/s41419-026-08554-y)
Supplement: Supplementary file 4 — Supplementary Figure legends [file 41419_2026_8554_MOESM4_ESM.docx]

**Supplementary Figure Legends**

**Supplementary Fig. S1. Quality control of protein samples for mass spectrometry analysis**

**A** Representative silver-stained SDS-PAGE gel of immunoprecipitated protein samples used for mass spectrometry: Lane 1: Protein molecular weight marker, Lane 2: Flag-NC IP eluate, Lane 3: Flag-FBXO6 IP eluate. These data demonstrate: Successful isolation of FBXO6-associated protein complexes; Appropriate sample integrity for downstream LC-MS/MS analysis. **B** CCK-8 assay at 48h shows mild inhibition by Flag-FBXO6 (~20% reduction vs. Flag-NC). **C** Prolonged 96h overexpression enhances inhibition (~30% reduction). **D-F** Cell viability assays demonstrate no toxicity between Flag-NC and sh-NC controls at 48h/96h. **G-H** Western blot quality control, uniform ITGB1 and GAPDH expression across controls confirms experimental consistency. **I-J** Repeat assays under standardized conditions show equivalent colony-forming capacity between sh-NC and Flag-NC controls. Data are presented as mean ± SD. ***p <* 0.01, ns (not signiﬁcant, P > 0.05) versus Flag-NC control group; Statistical analysis was performed using one-way ANOVA with Tukey´s post hoc test in B-H. Data in J was analyzed using unpaired Student´s t-test.

**Supplementary Fig. S2. Clinical validation of FBXO6 and ITGB1 expression in colorectal cancer patient tissues**

**A** qRT-PCR analysis of FBXO6 and ITGB1 transcripts in paired CRC tumours and adjacent normal tissues (n = 10). No significant differences were detected for either gene. **B** Representative western blot and densitometric quantification of FBXO6 and ITGB1 proteins (normalised to GAPDH) in the same tissue pairs (n = 10 per group). ITGB1 protein was significantly elevated in tumours, whereas FBXO6 showed no change. **C** Representative immunohistochemistry (IHC) images and corresponding H-score quantification of FBXO6 and ITGB1 in CRC versus normal tissues (n = 3). Consistent with the western blot data, ITGB1 staining intensity was markedly higher in tumour regions, while FBXO6 remained unchanged (ns). Scale bars,100 μm. Data are presented as mean ± SD; statistical significance was determined with an unpaired two-tailed Student’s t-test (**p ＜* 0.05; ns, not significant).

**Supplementary Fig. S3. In vivo validation of ITGB1’s pro-tumorigenic role in CRC xenograft models**

**A-B** Representative appearance of tumors derived from nude mice. NC: Negative control group; OE-ITGB1: ITGB1-overexpressing HCT116 cell group; sh-ITGB1: ITGB1-knockdown HCT116 cell group; FLAG-FBXO6+ITGB1: FBXO6 and ITGB1 co-overexpressing HCT116 cell group, n=4 mice per group. Observed for 25 days, with tumor volume measured every 5 days. The tumor growth curves showed that the OE-ITGB1 group had larger tumors than the NC group, while the sh-ITGB1 group had smaller tumors than the NC group. The FLAG-FBXO6+ITGB1 group rescued the tumor-enhancing trend observed in the OE-ITGB1 group. Data are presented as mean ± SD. ***p* < 0.01 compared to the control group; statistical analysis was performed using one-way ANOVA followed by Tukey’s post hoc test.
