# Supplementary material for: FBXO6 regulates colon cancer migration and invasion via ITGB1 ubiquitination and downstream signaling
Source: Cell Death Dis. 2026 Mar 19;17(1):324. doi: 10.1038/s41419-026-08554-y (PMC13039278; doi:10.1038/s41419-026-08554-y)

Fig. 1D

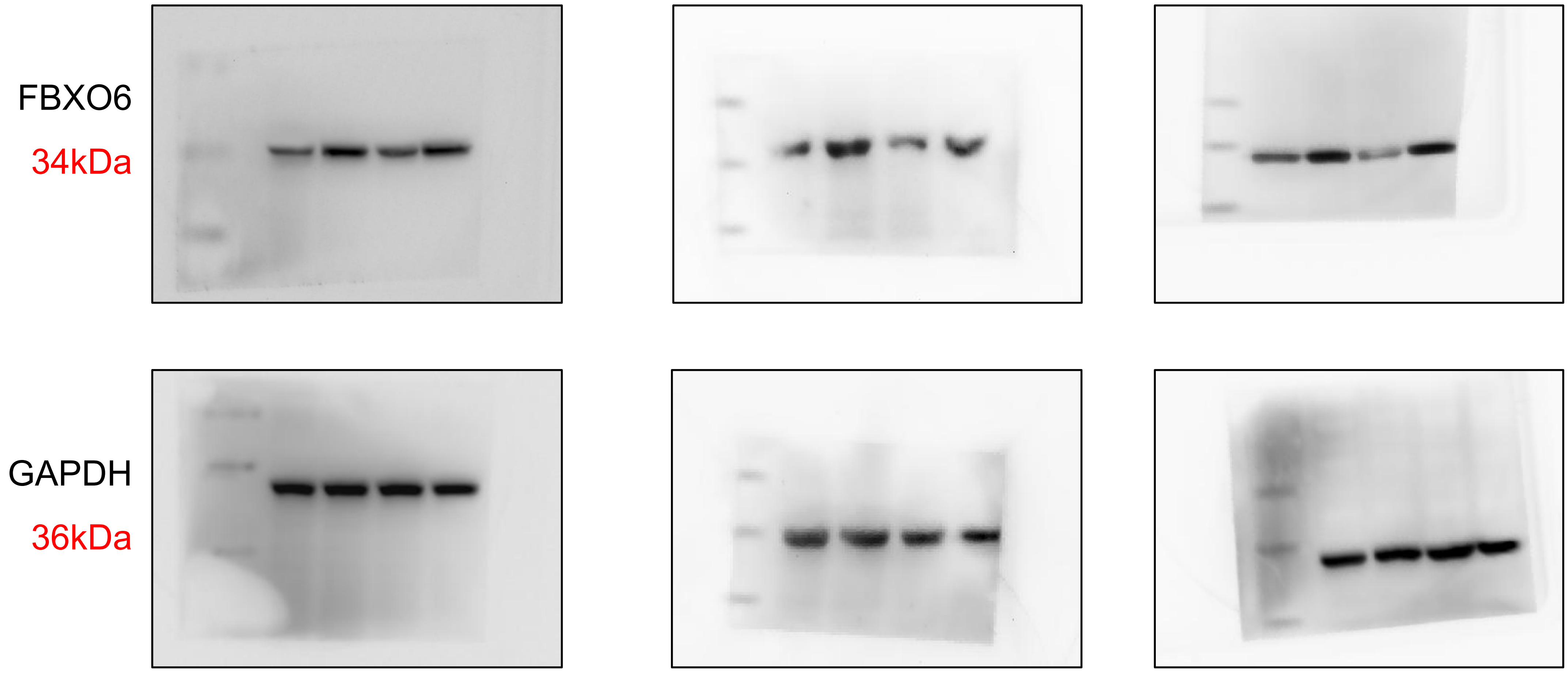

Fig. 2B

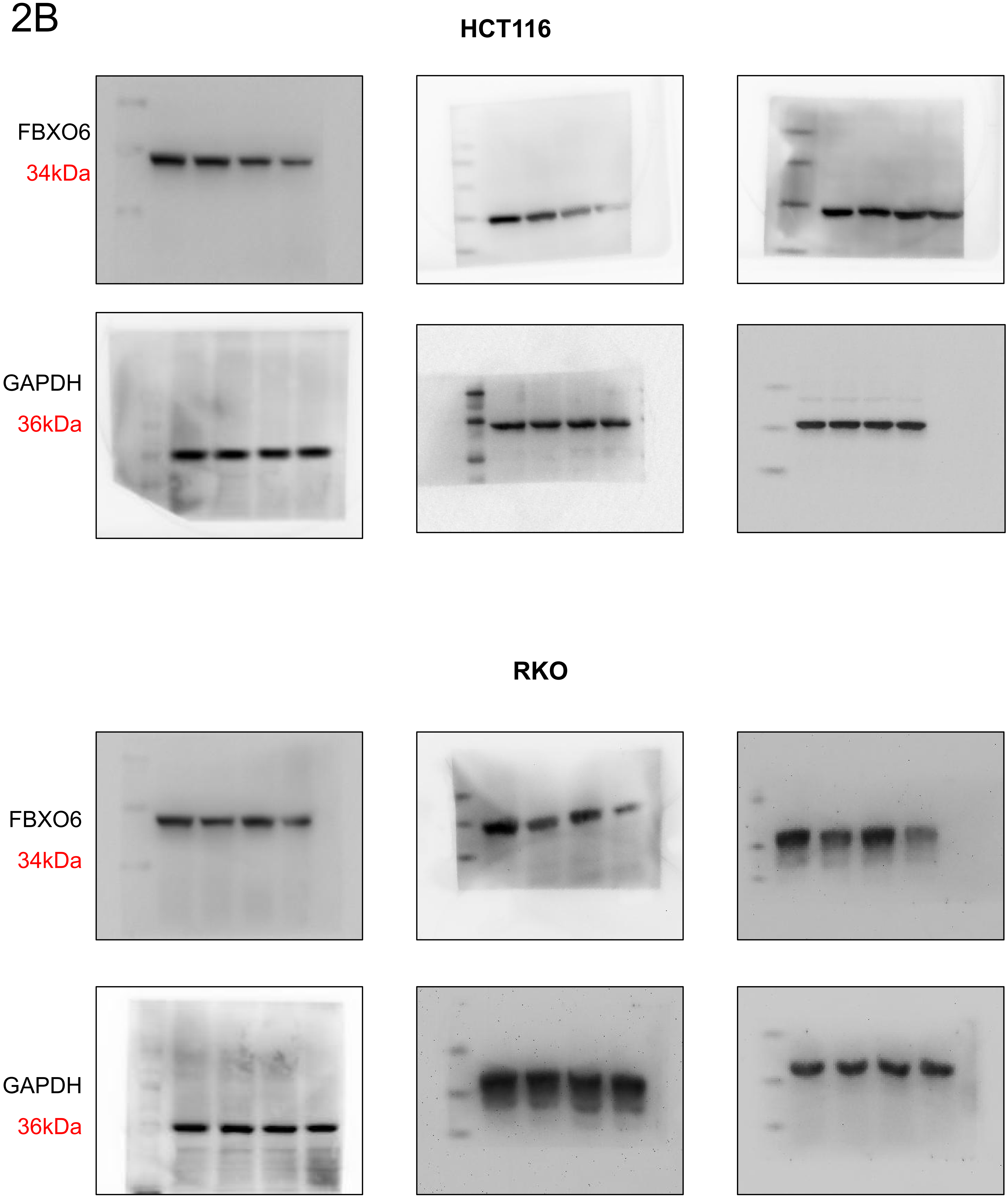

Fig. 3A

FLAG

1.0kDa

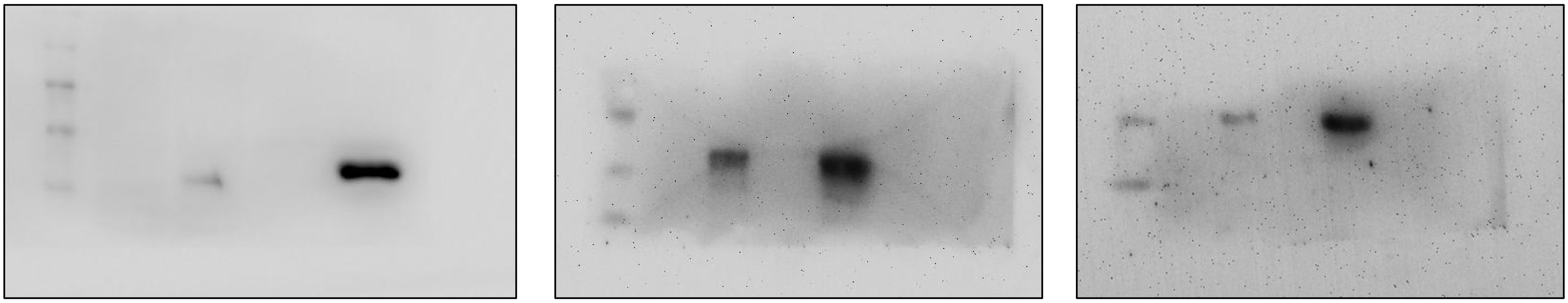

FBXO6

34kDa

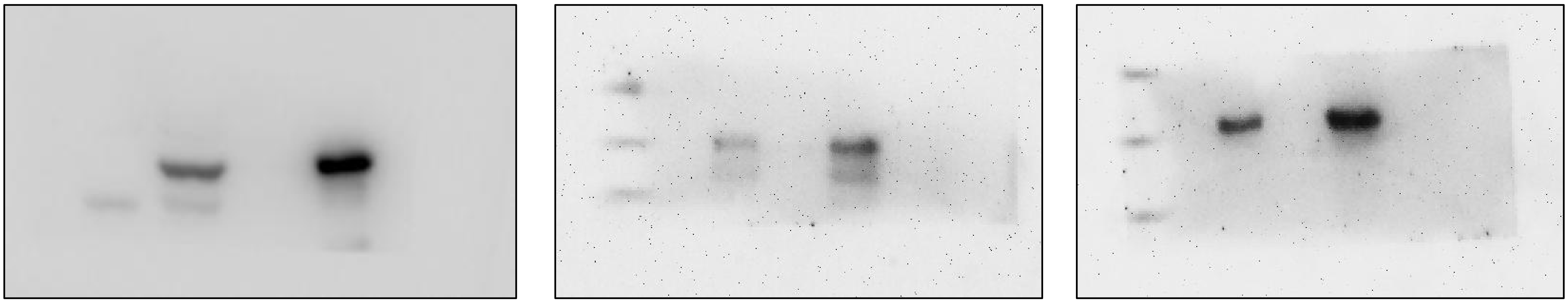

GAPDH

36kDa

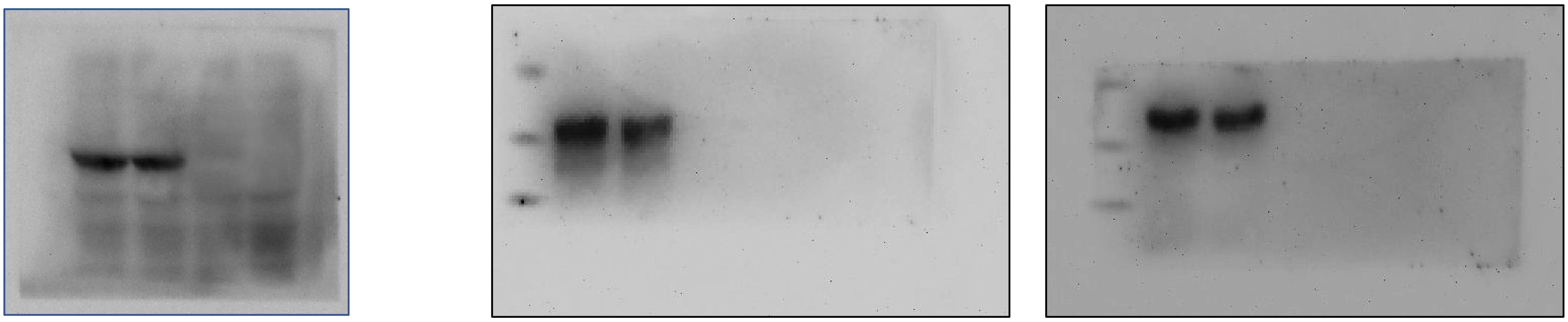

Fig. 4A

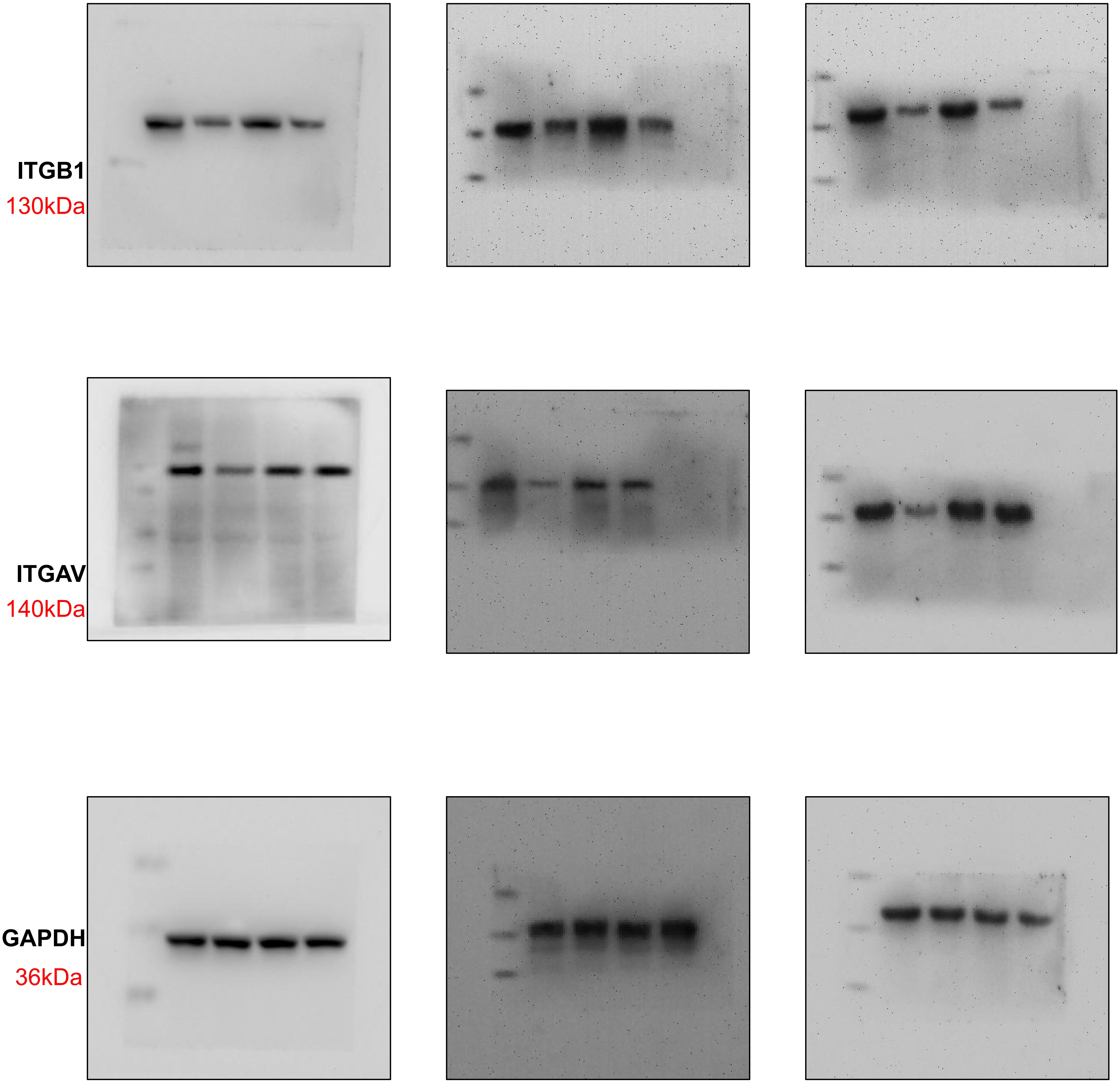

Fig. 4E

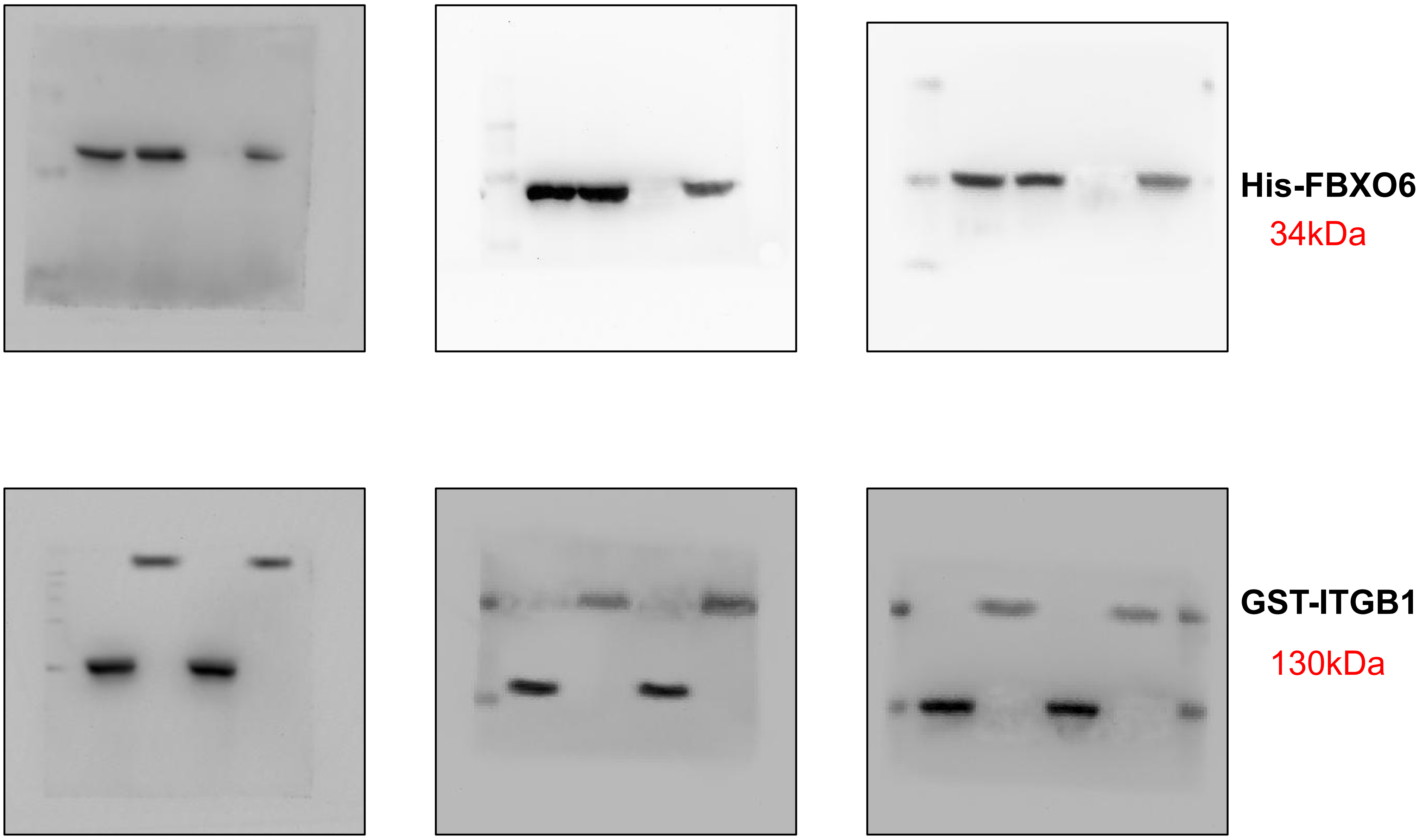

Fig. 4F-HCT116

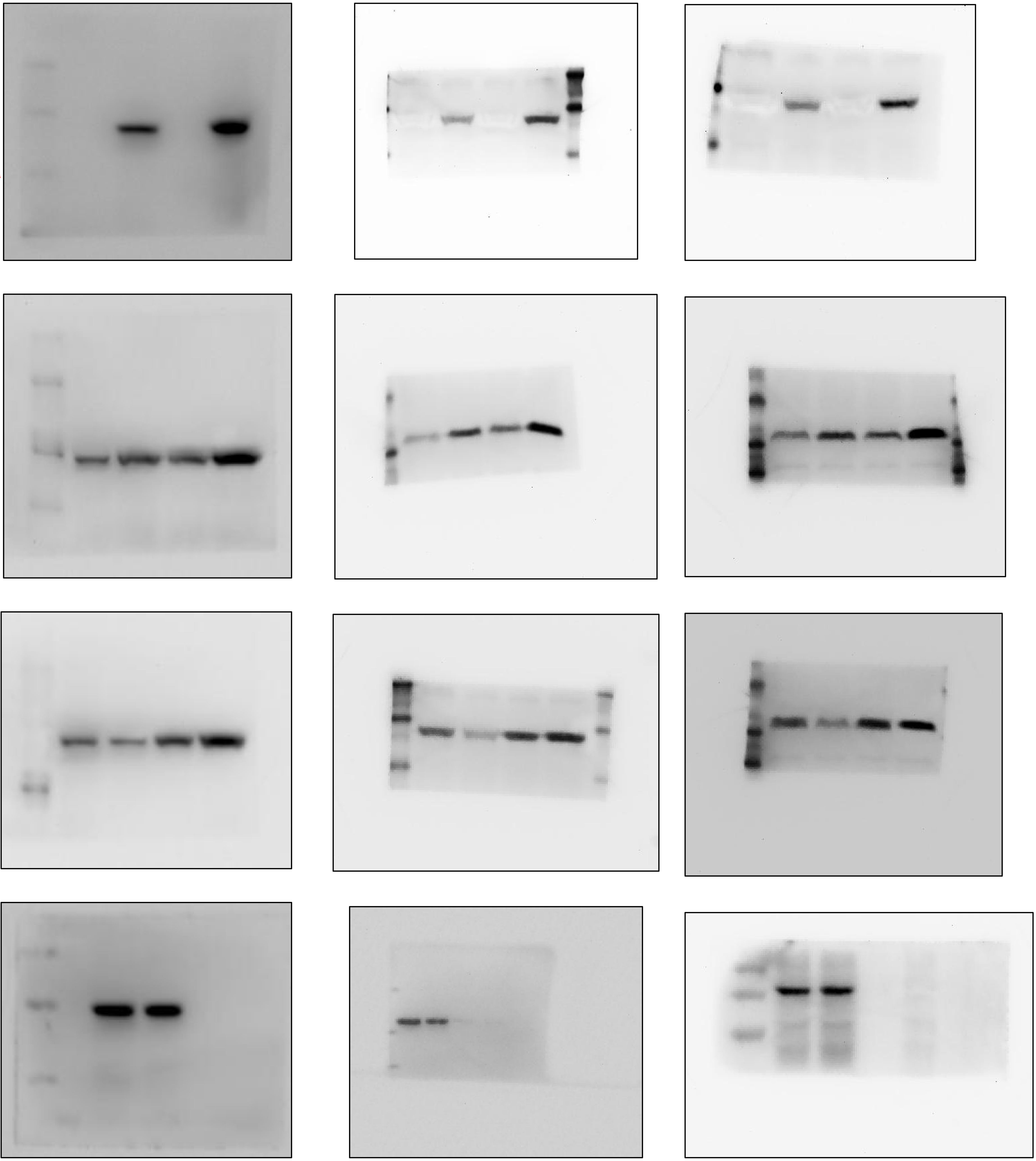

FLAG
1.0kDa

FBXO6
34kDa

ITGB1
130kDa

GAPDH
36kDa

Fig. 4F-RKO

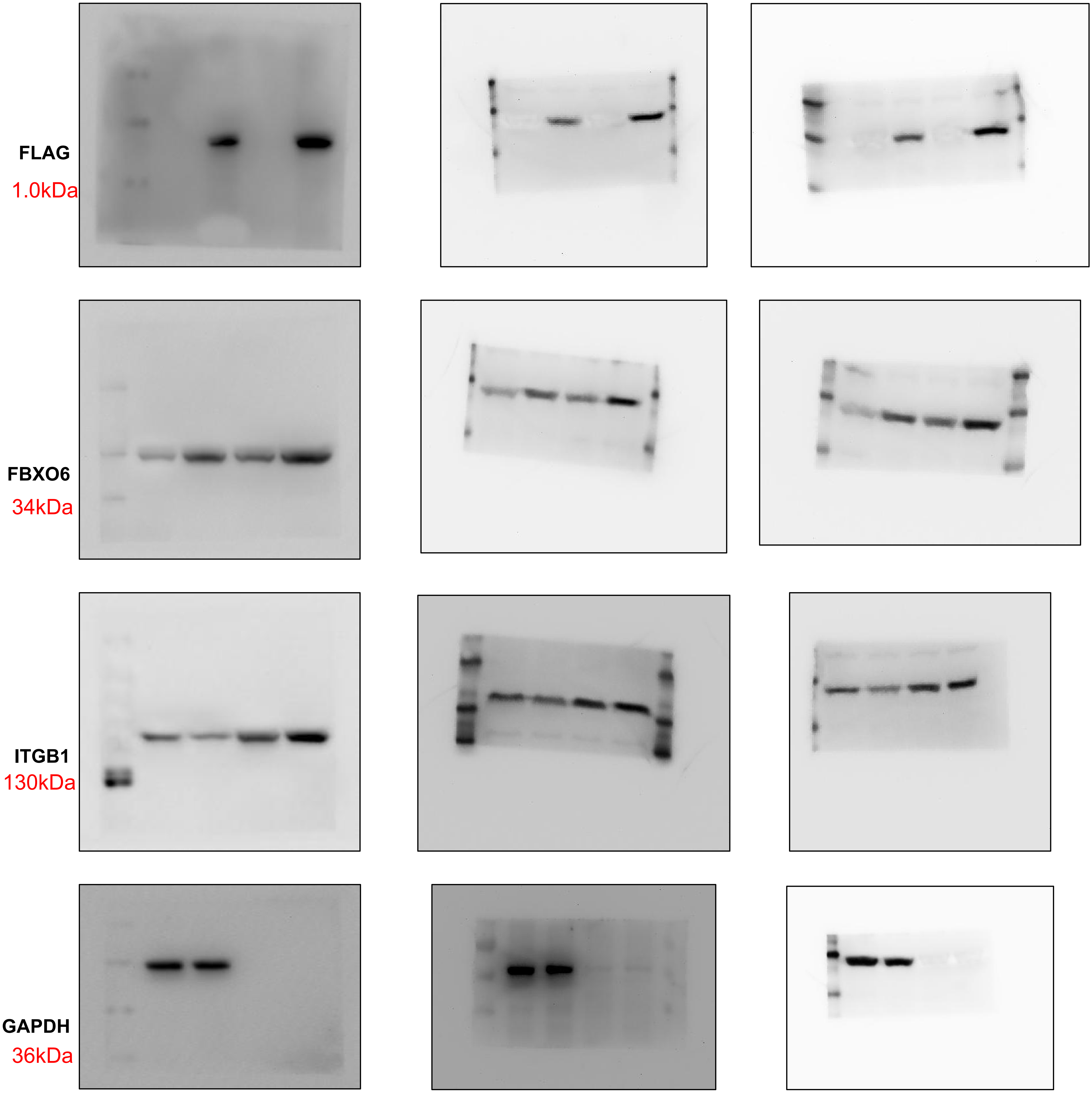

Fig. 5A

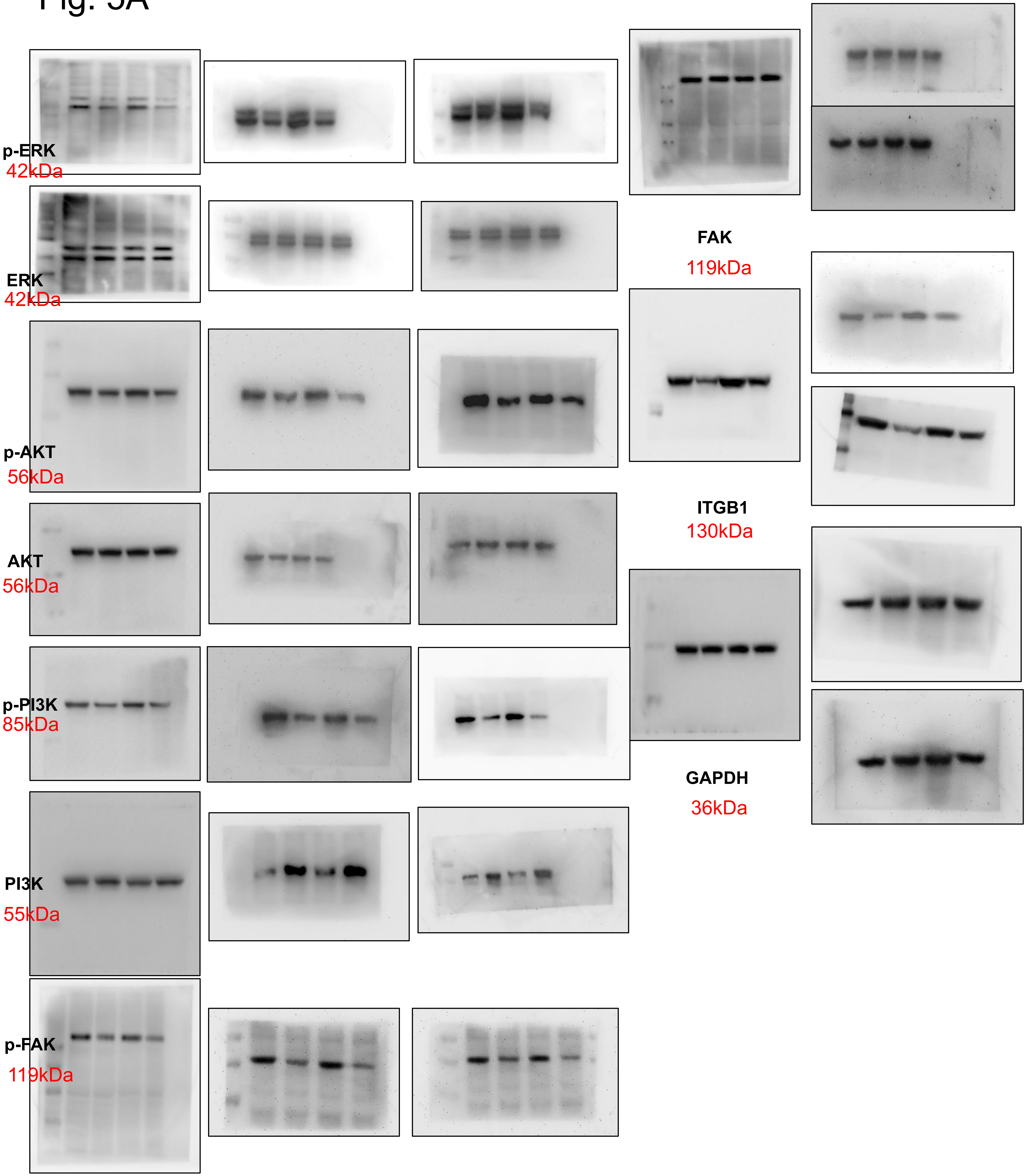

p-ERK
42kDa
ERK
42kDa
p-AKT
56kDa
AKT
56kDa
p-PI3K
85kDa
PI3K
55kDa
p-FAK
119kDa
FAK
119kDa
ITGB1
130kDa
GAPDH
36kDa

Fig. 5G

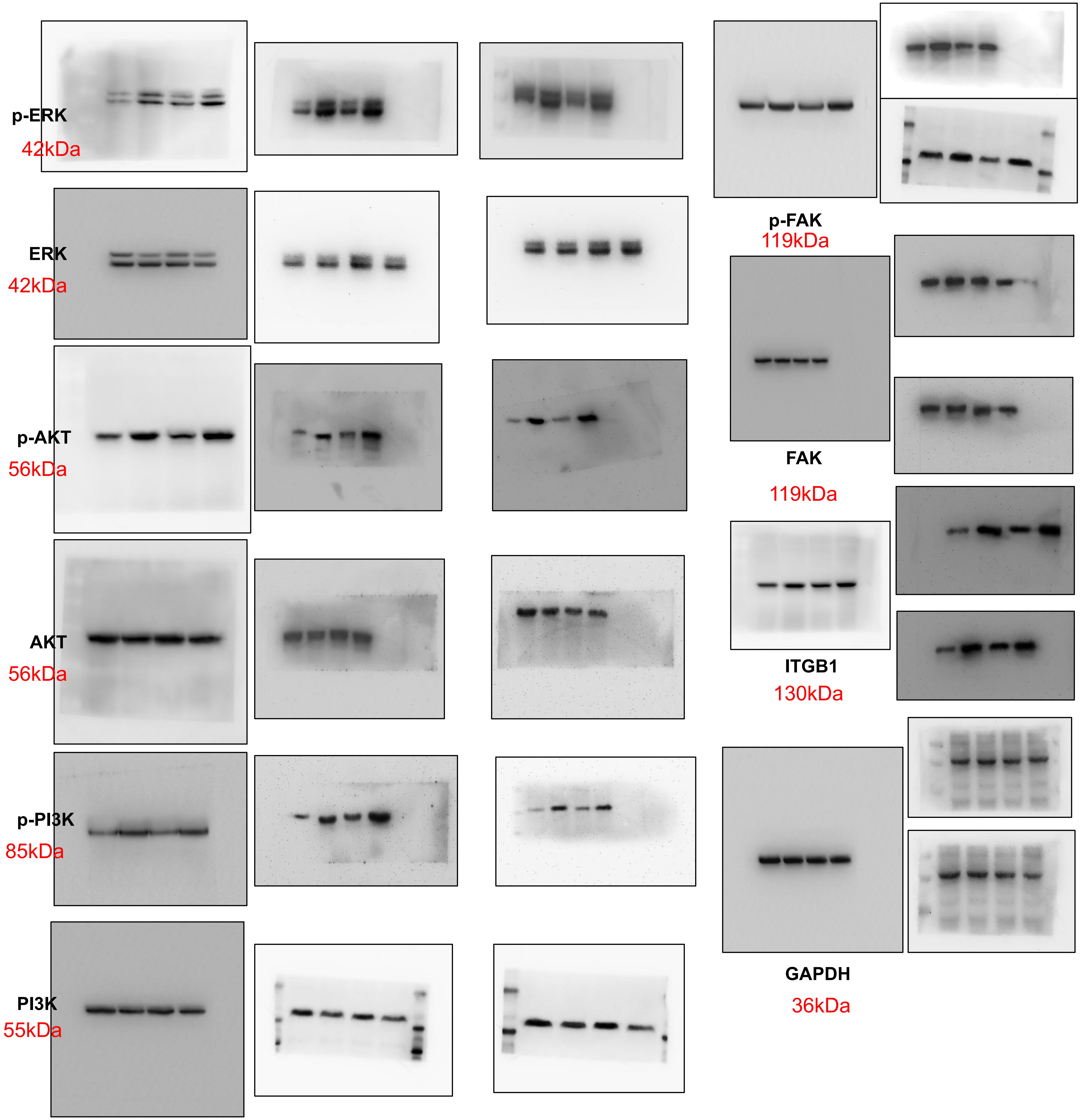
p-ERK
42kDa
ERK
42kDa
p-AKT
56kDa
AKT
56kDa
p-PI3K
85kDa
PI3K
55kDa
p-FAK
119kDa
FAK
119kDa
ITGB1
130kDa
GAPDH
36kDa

Fig. 5M

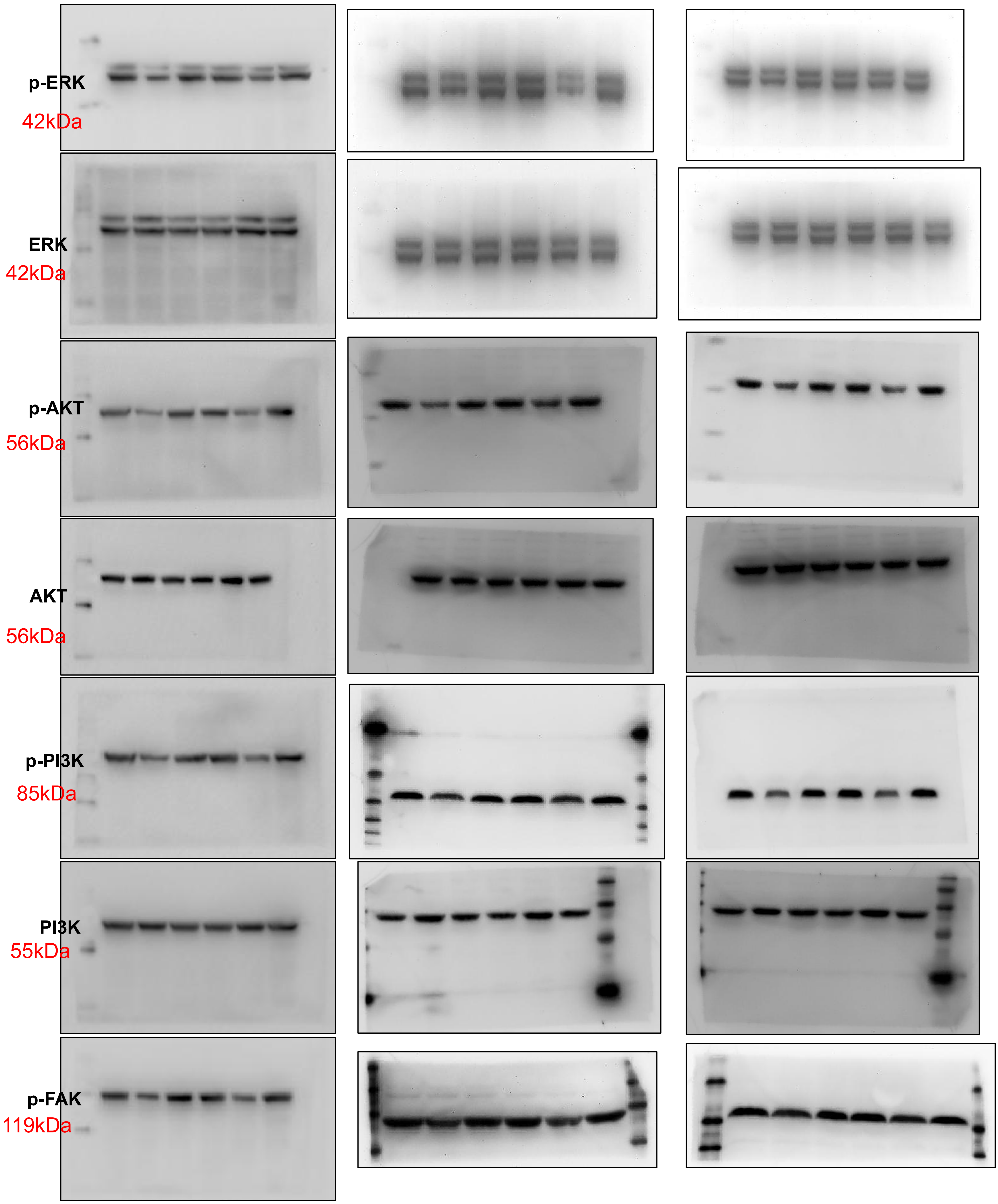
p-ERK
42kDa
ERK
42kDa
p-AKT
56kDa
AKT
56kDa
p-PI3K
85kDa
PI3K
55kDa
p-FAK
119kDa

Fig. 5M

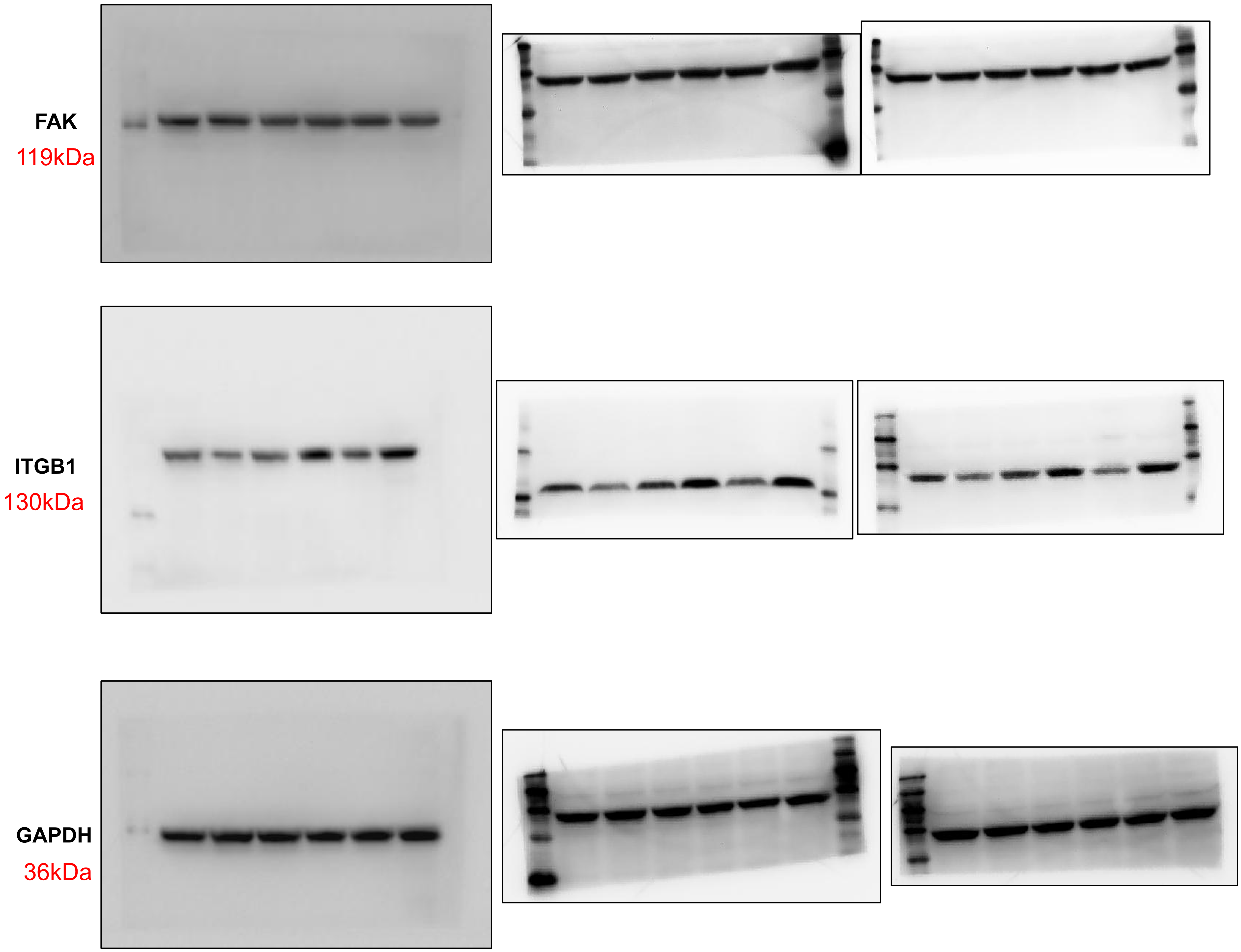

Fig. 6A

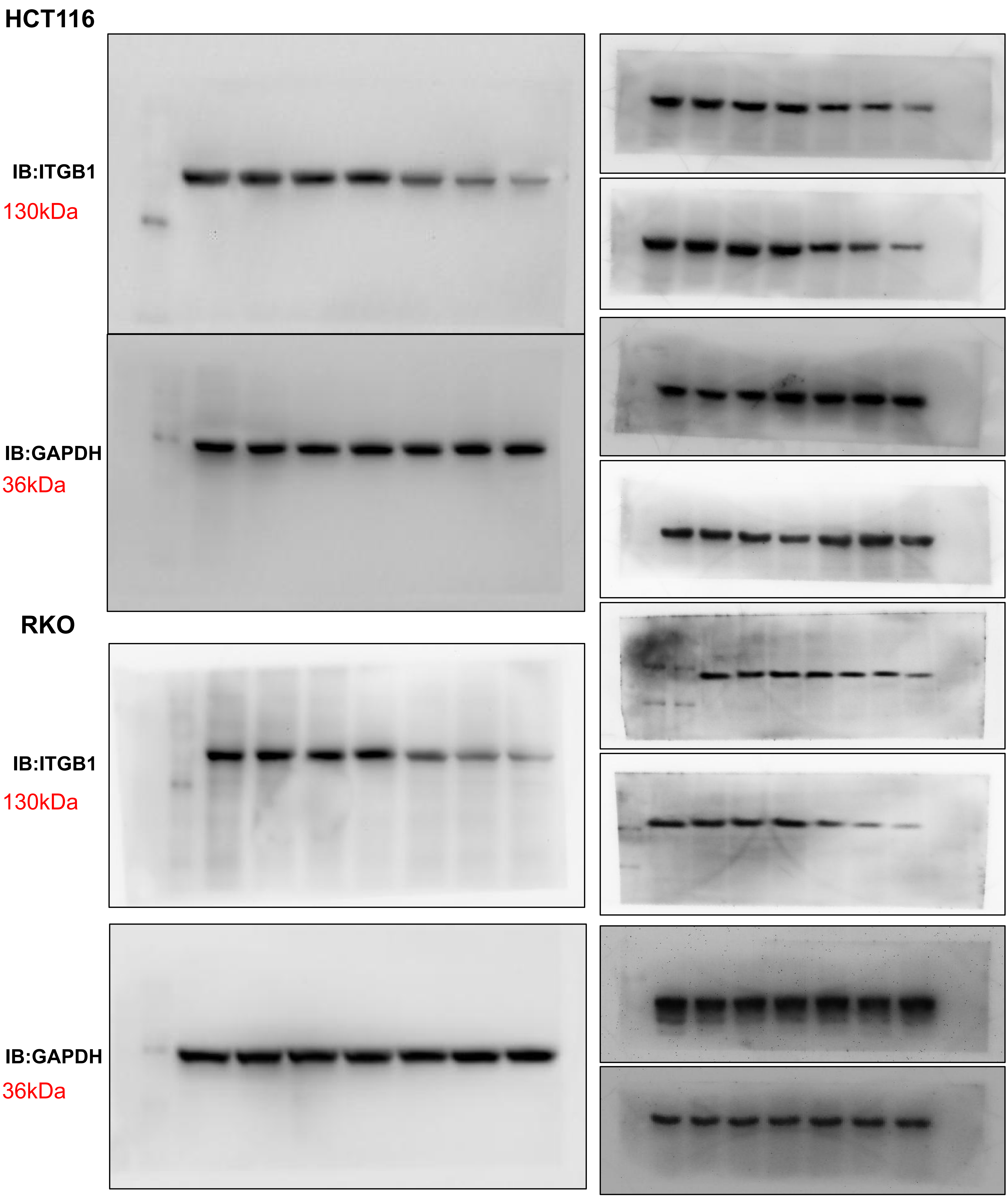

HCT116
IB:ITGB1
130kDa
IB:GAPDH
36kDa
RKO
IB:ITGB1
130kDa
IB:GAPDH
36kDa

Fig. 6B

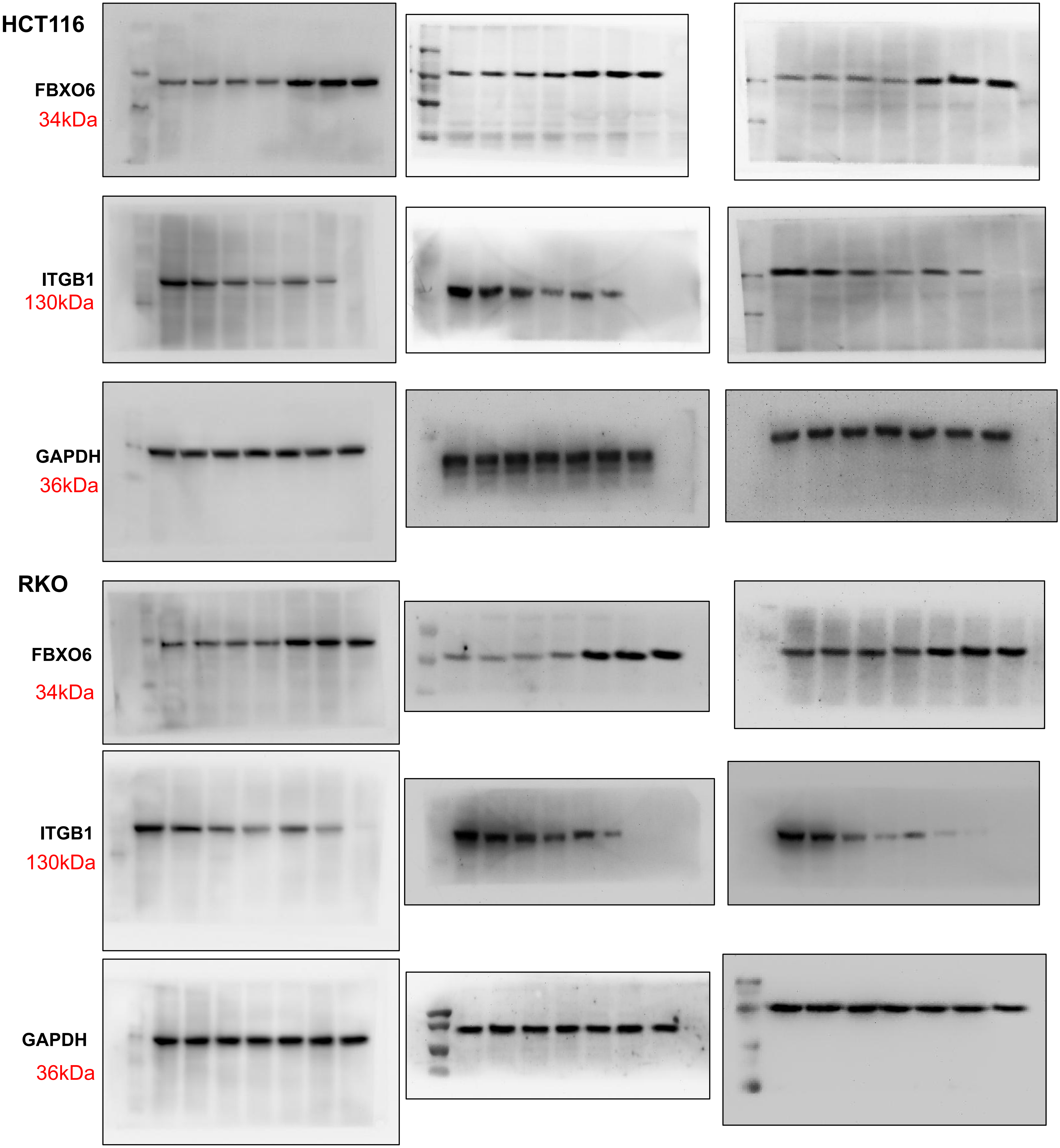

HCT116
FBXO6
34kDa
ITGB1
130kDa
GAPDH
36kDa
RKO
FBXO6
34kDa
ITGB1
130kDa
GAPDH
36kDa

Fig. 6C

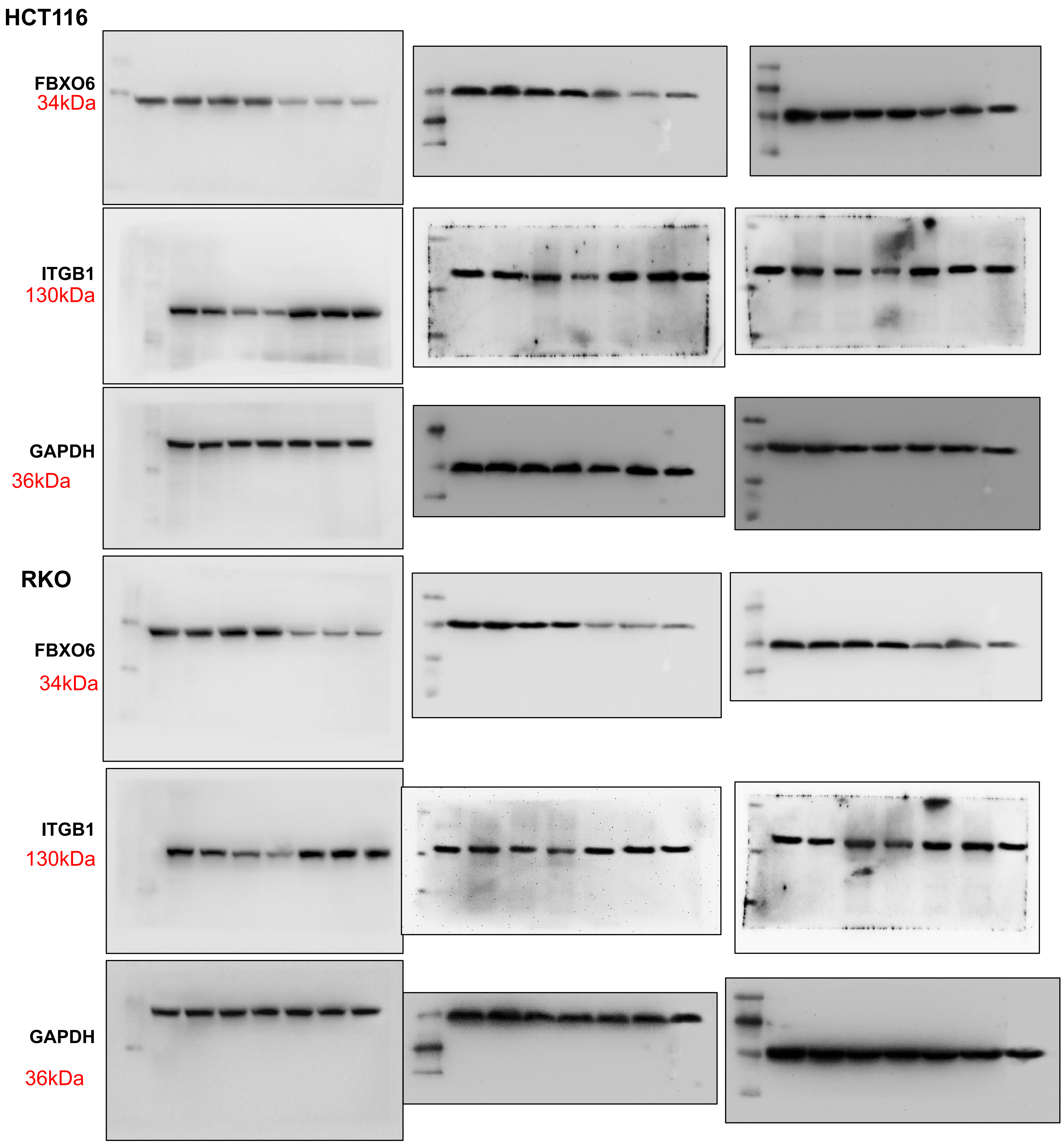
HCT116
FBXO6
34kDa
ITGB1
130kDa
GAPDH
36kDa
RKO
FBXO6
34kDa
ITGB1
130kDa
GAPDH
36kDa

Fig. 6D

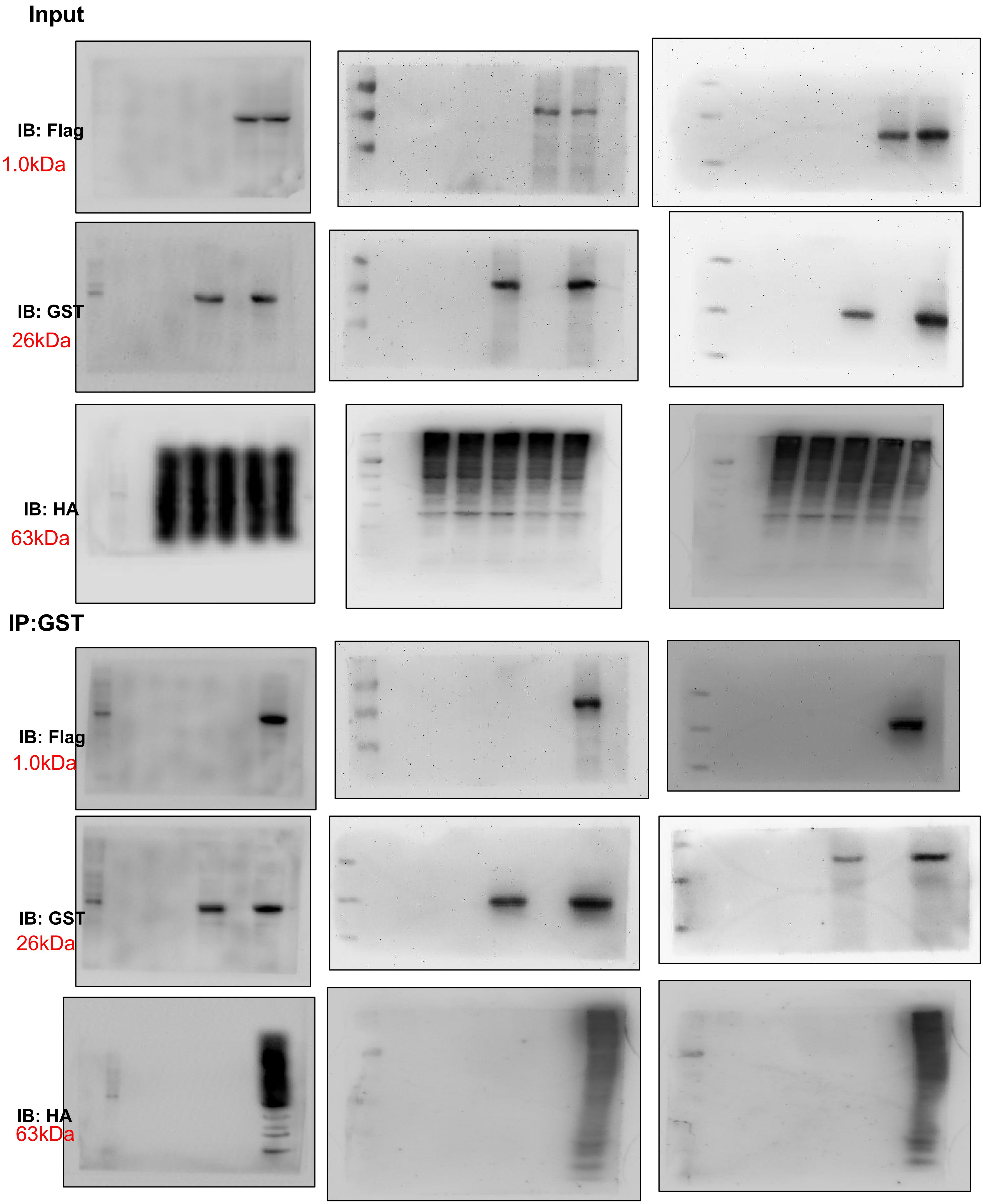
Input
IB: Flag
1.0kDa
IB: GST
26kDa
IB: HA
63kDa
IP:GST
IB: Flag
1.0kDa
IB: GST
26kDa
IB: HA
63kDa

Fig. 7F-HCT116

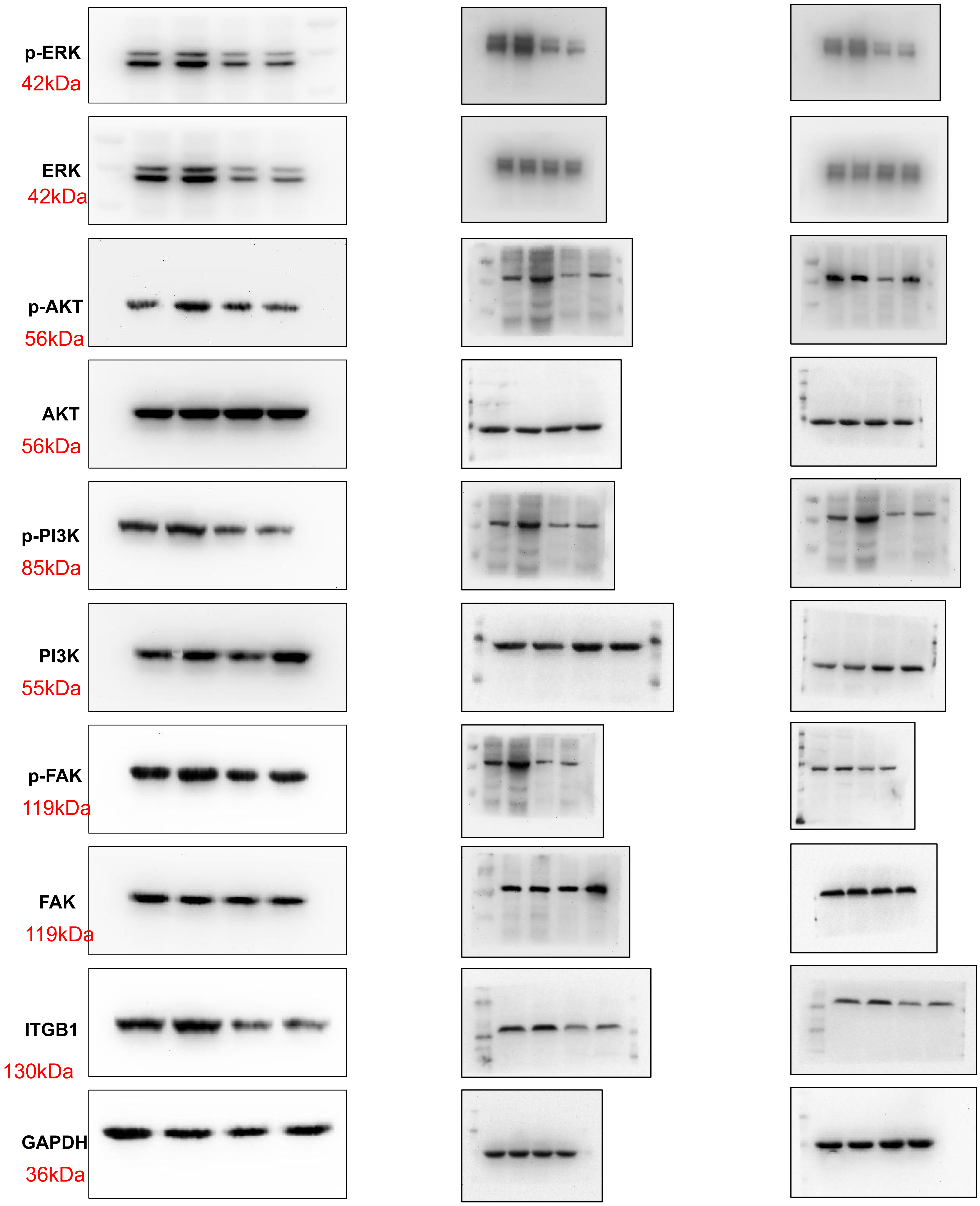

Fig. 7F-RKO

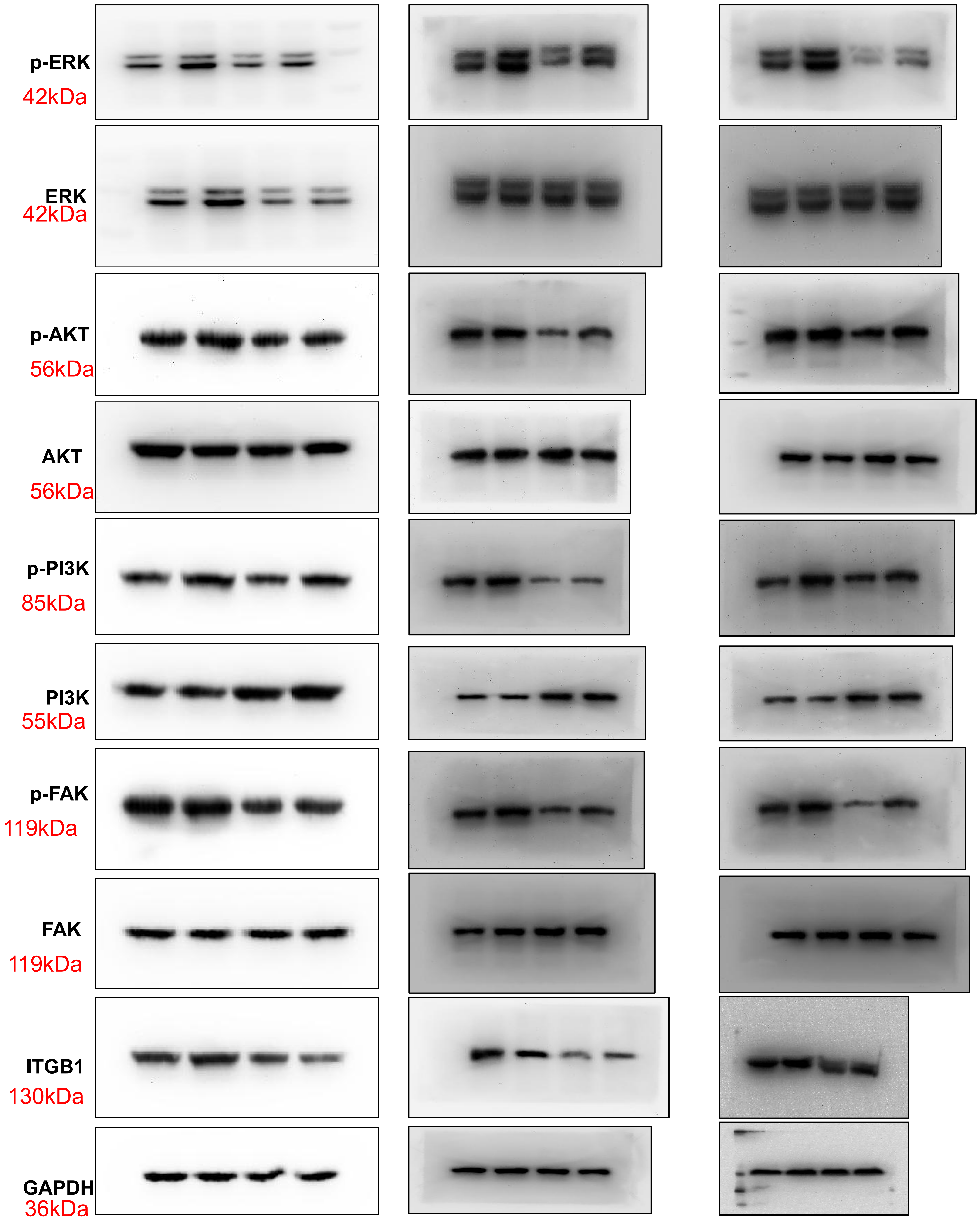

p-ERK
42kDa
ERK
42kDa
p-AKT
56kDa
AKT
56kDa
p-PI3K
85kDa
PI3K
55kDa
p-FAK
119kDa
FAK
119kDa
ITGB1
130kDa
GAPDH
36kDa

Fig. 8E

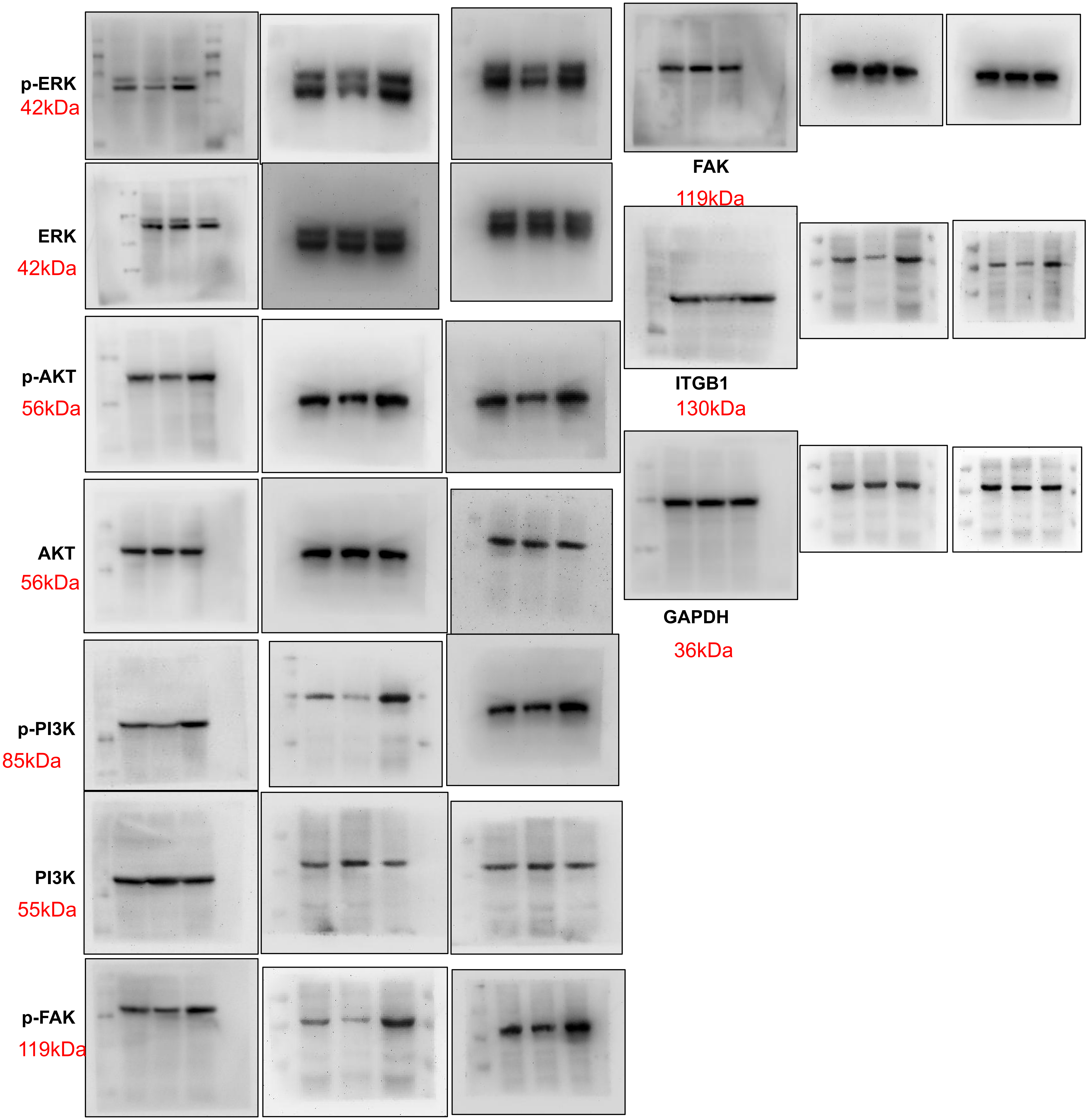
p-ERK
42kDa
ERK
42kDa
p-AKT
56kDa
AKT
56kDa
p-PI3K
85kDa
PI3K
55kDa
p-FAK
119kDa
FAK
119kDa
ITGB1
130kDa
GAPDH
36kDa

Fig. S1G

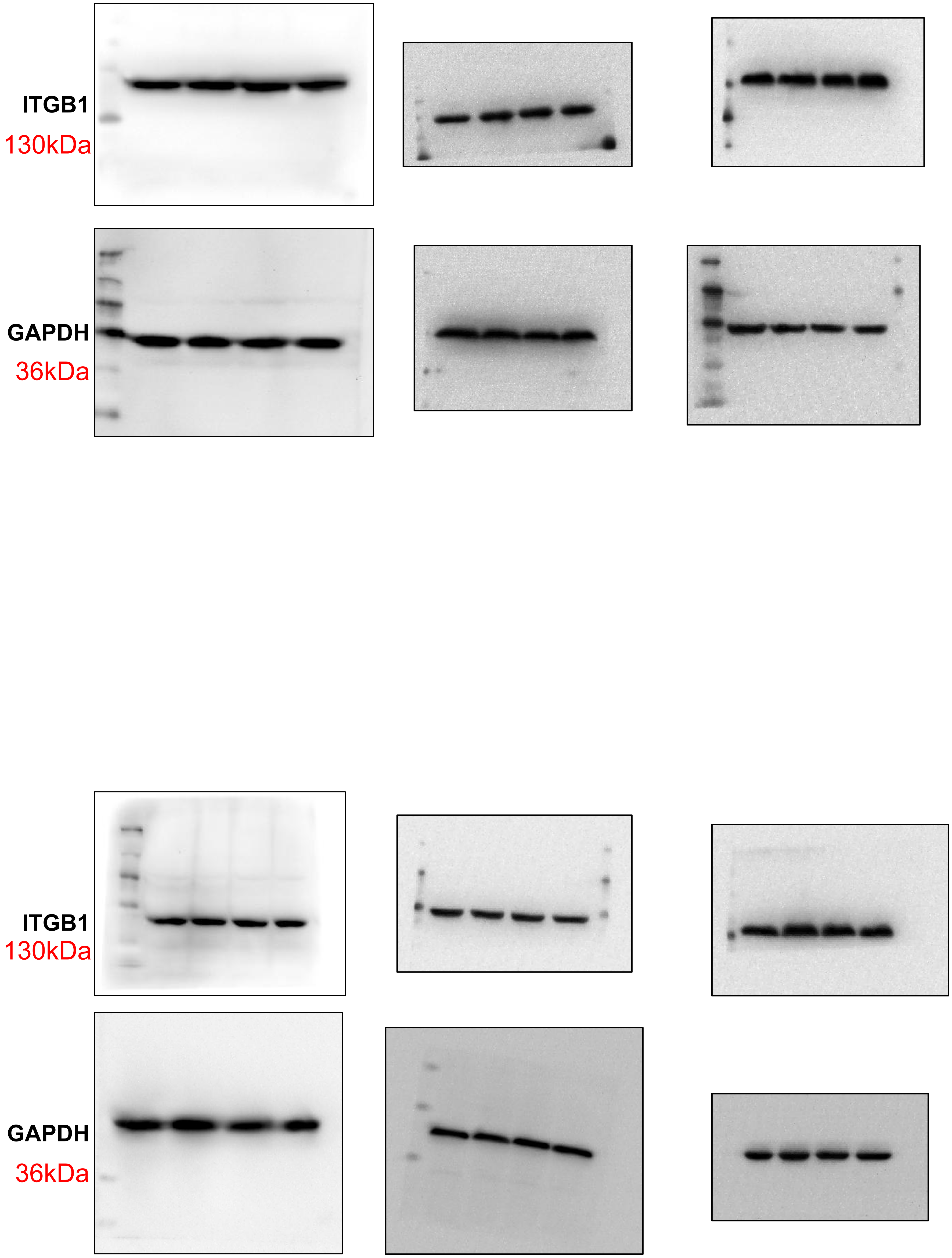

Fig. S2B

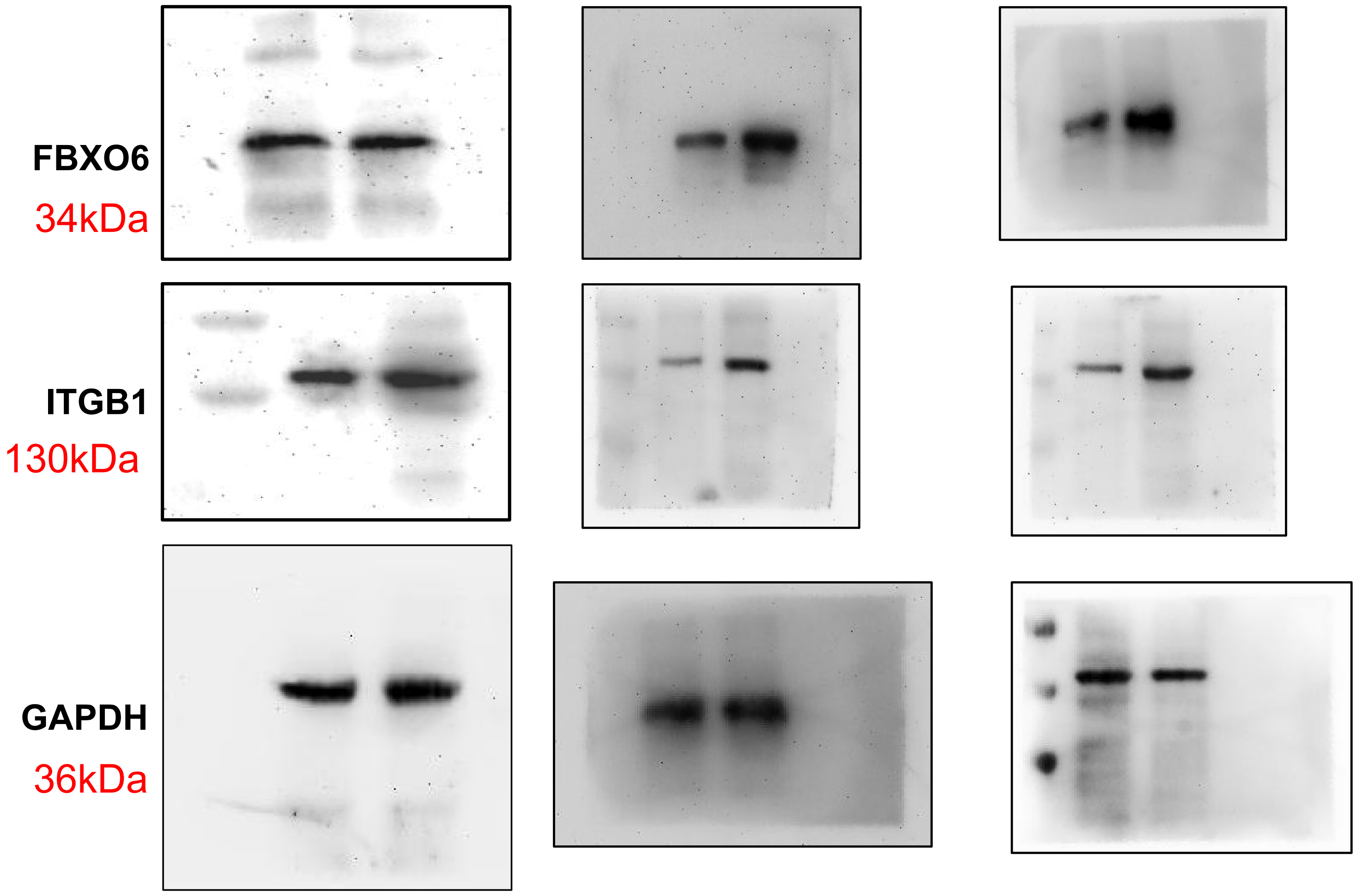

Supplement: Supplementary file 5 — Supplemental Material--WB [file 41419_2026_8554_MOESM5_ESM.pdf]
